# Exogenous γ-aminobutyric acid (GABA) affects pollen tube growth via modulating putative Ca^2+^-permeable membrane channels and is coupled to negative regulation on glutamate decarboxylase

**DOI:** 10.1093/jxb/eru171

**Published:** 2014-05-05

**Authors:** Guang-Hui Yu, Jie Zou, Jing Feng, Xiong-Bo Peng, Ju-You Wu, Ying-Liang Wu, Ravishankar Palanivelu, Meng-Xiang Sun

**Affiliations:** ^1^Department of Cell and Development Biology, College of Life Science, State Key Laboratory of Plant hybrid rice, Wuhan University, Wuhan 30072, China; ^2^Key Laboratory for Biotechnology of the State Ethnic Affairs Commission, Hubei provincial Key laboratory for protection and application of special plants in Wuling Area of China, College of Life Sciences, South-Central University for Nationalities, Wuhan 430074, China; ^3^State key laboratory of Virology, College of Life Science, Wuhan University, Wuhan 430072, China; ^4^College of Horticulture, State key laboratory of crop genetics and germplasm enhancement, Nanjing Agriculture University, Nanjing 210095, China; ^5^School of Plant Sciences, University of Arizona, Tucson, AZ 85721-0036, USA

**Keywords:** γ-Aminobutyric acid, Ca^2+^-permeable channel, cell–cell communication, glutamate decarboxylase, *Nicotiana tabacum*, pollen tube.

## Abstract

γ-Aminobutyric acid (GABA) is implicated in pollen tube growth, but the molecular and cellular mechanisms that it mediates are largely unknown. Here, it is shown that exogenous GABA modulates putative Ca^2+^-permeable channels on the plasma membranes of tobacco pollen grains and pollen tubes. Whole-cell voltage-clamp experiments and non-invasive micromeasurement technology (NMT) revealed that the influx of Ca^2+^ increases in pollen tubes in response to exogenous GABA. It is also demonstrated that glutamate decarboxylase (GAD), the rate-limiting enzyme of GABA biosynthesis, is involved in feedback controls of Ca^2+^-permeable channels to fluctuate intracellular GABA levels and thus modulate pollen tube growth. The findings suggest that GAD activity linked with Ca^2+^-permeable channels relays an extracellular GABA signal and integrates multiple signal pathways to modulate tobacco pollen tube growth. Thus, the data explain how GABA mediates the communication between the style and the growing pollen tubes.

## Summary

This work reveals how tobacco pistil and style may communicate with pollen tubes and regulate their growth during fertilization via the γ-aminobutyric acid–Ca^2+^-permeable channel–glutamate decarboxylase–calmodulin signalling pathway.

## Introduction

Fertilization during reproduction in flowering plants begins when pollen grains land on a compatible stigma. Following this, complex signal transduction networks mediate multiple developmental events including the directional outgrowth of the pollen tube, elongation of the pollen tube over long distances in the style tract, and delivery of sperm cells to the embryo sac within the ovule to complete fertilization (Sauter, 2009). Multiple signals are involved in the regulation of these events (Palanivelu and Sukamoto, 2012). For example, γ-aminobutyric acid (GABA), which acts as a neurotransmitter in animals, also functions in pollen tube migration, as discovered in *Arabidopsis* (Palanivelu *et al.*, 2003). In plants, GABA is a ubiquitous molecule that acts in the responses to a variety of environmental signals, including hypoxia, acidosis, mechanical stress, and cold stress (Baum *et al.*, 1996; Shelp *et al.*, 1999; Bouché and Fromm, 2004; Renaul *et al.*, 2011). The pathway of GABA metabolism (GABA shunt pathway) has been elucidated. GABA synthesis from glutamate is catalysed by cytosolic glutamate decarboxylase (GAD; EC 4.1.1.15); GABA is then oxidized in mitochondria via two successive steps, catalysed by GABA transaminase and succinic semialdehyde dehydrogenase (SSADH; EC 1.2.1.16), to form succinate, which then enters the tricarboxylic acid cycle (Shelp *et al.*, 1999).

*Arabidopsis pop2* mutants are defective in the gene that codes for GABA transaminase (GABA-T, POP2; EC 2.6.1.19). Loss of GABA transaminase function in *pop2* mutants results in GABA accumulation in pistils, consistent with its role in metabolizing GABA in the GABA shunt pathway, and *pop2* pollen tube growth is arrested or misguided in *pop2* pistils (Palanivelu *et al.*, 2003). The GABA accumulation in *pop2* pistils abolishes the increasing GABA gradient from the stigma to the ovule micropyle that is present in wild-type pistils. These results led to the suggestion that loss of the GABA gradient in *pop2* pistils results in abnormal pollen tube growth. However, the role of GABA in the regulation of pollen tube growth and how it mediates pollen tube–pistil interactions remain largely unknown.

In plants, GAD is involved in GABA accumulation in response to environmental stresses. Plant GADs possess an autoinhibitory domain in the C-terminal segment; this domain restrains GAD activity under normal growth conditions. The inhibition function can be removed by the binding of the Ca^2+^/calmodulin (CaM) complex to this domain (Chen *et al.*, 1994; Snedden *et al.*, 1996). Thus, when the steady-state level of cytosolic Ca^2+^ increases, GAD inhibition is rapidly released upon binding to Ca^2+^/CaM, leading to immediate synthesis of GABA. It is well known that Ca^2+^ plays a significant role in the regulation of pollen tube growth (Franklin-Tong, 1999). Previous work showed that CaM is an important intermediary in Ca^2+^ signalling in growing pollen tubes (Ma and Sun, 1997) and is also a modulator of GAD activity (Baum *et al.*, 1996; Shelp *et al.*, 1999). Therefore, examining the participation of Ca^2+^ in the regulation of GAD activity and its CaM binding ability is essential to understand the functions of GAD/GABA in pollen tube growth.

The rapid tip growth of tobacco pollen tubes serves as a good *in vitro* model system to study signal transduction in plants. Many proteins are involved in the regulation of pollen tube tip growth and vesicle trafficking, including a heterotrimeric G protein (Ma *et al.*, 1999), phospholipase C (PLC) (Dowd *et al.*, 2006; Helling *et al.*, 2006), phospholipase D (PLD) (Potocky *et al.*, 2003), mitogen-activated protein kinases (MAPK) (Wilson *et al.*, 1995), and the Rop/RacGTPase molecular switches and related regulatory molecules (Cheung *et al.*, 2003; Klahre *et al.*, 2006; Klahre and Kost, 2006; Lavy *et al.*, 2007; Szumlanski and Nielsen, 2009). To understand the function of GABA in pollen tube growth, it is therefore necessary to clarify the role of GABA in the complex network comprising these proteins and the pathway(s) in which GABA may be involved.

Here, it is demonstrated that exogenous GABA is involved in the regulation of pollen tube growth via activation of Ca^2+^-permeable channels at low concentration and inhibition at high concentration. It is also shown that GAD plays a critical role in regulating tobacco pollen tube growth by functioning as a downstream regulator of Ca^2+^/CaM and feedback control on Ca^2+^-permeable channels. The regulatory relationships of these molecules to the aforementioned genes were also investigated to identify the GABA signalling pathway in pollen tubes.

## Materials and methods

### Chemicals

All chemicals were purchased from Sigma unless otherwise stated.

### Plant growth conditions and pollen germination

Plants of *Nicotiana tabacum* SR1 were grown at 22 °C in a greenhouse at Wuhan University, China, under a 16h photoperiod. Fresh pollen of flowers at stage 12 were cultivated *in vitro* in germination medium (GM) at 25 °C in darkness. GM contained 1.0mM CaCl_2_, 1.0mM KCl, 0.8mM MgSO_4_, 1.6mM H_3_BO_4_, 30.0 μM CuSO_4_, 5.0mM MES, and 20% sucrose, and the pH was adjusted to 5.8 with 1.0M TRIS. The images of growing pollen tubes were collected with the capture function of a Leica DMIRE 2 inverted microscope equipped with a CCD camera (RTE/CCD-1300-Y/HS, Roper Scientific Co.).

### Pollen tube measurement

The time-lapse images of pollen tube growth were captured in 1h intervals for later measurement. The tube length was defined as the distance from the central point of a pollen grain to the tip of its pollen tube. The average length of 40 randomly selected pollen tubes was regarded as the pollen tube length at each time point. Different concentrations of 3-mercaptopropionic acid (3-MPA) were used within the range of 50 μM to 5.0mM. After treatment, pollen tubes were fixed in Carnoy’s solution (ethanol:glacial acetic acid, 3:1, v/v) supplemented with 20% sucrose. Lengths of pollen tubes were measured using the measure function of Metamorph Software, which is capable of measuring the length of curved pollen tubes. Each treatment was a randomized block design (RBD) with three replicates.

### Pollen tube protoplast isolation

Tobacco pollen tube protoplasts were prepared as follows (Yu *et al.*, 2006). The collected pollen grains were first germinated in GM [1.0mM KNO_3_, 1.0mM Ca (NO_3_)_2_, 1.0mM MgSO_4_, 1.0mM H_3_BO_3_, and 20% sucrose, pH 5.8] at 25 °C. When the pollen tubes had emerged and were vigorously growing, they were transferred into an enzyme solution containing 1% cellulose (Onozuka R-10) and 1% pectinase (Merck) dissolved in bath solution, and incubated at 28 °C for 1h to release pollen protoplasts.

### Electrophysiology

Whole-cell voltage-clamp experiments were carried out at 20 °C using an EPC 10 patch clamp amplifier (HEKA Elekt-ronik, Lambrecht, Germany) interfaced to a computer running acquisition and analysis software (Pulse). A multichannel microperfusion system (MPS-2; INBIO Inc., Wuhan, China) was used to exchange the external recording solution. The membrane was held at a holding potential of 0 mV and liquid junction potentials were corrected according to a previous method (Neher, 1992; Wu *et al.*, 2011). Pipettes were pulled from glass capillaries in two stages, with pipette resistance ranging from 20 MΩ to 30 MΩ when filled with pipette solutions. The standard solution used for ICa measurements contained 50mM CaCl_2_, 2.5mM MES, pH 5.8 adjusted with TRIS, and the pipette solution contained 0.1mM CaCl_2_, 4mM Ca(OH)_2_, 10mM HEPES, 10mM EGTA, 2mM MgATP, pH 7.2. The osmolalities of the pipette and bath solutions were adjusted to 1614 mosmol kg^–1^ with d-sorbitol. When GABA was added to the bathing solution, spontaneous ICa-like currents were recorded on the pollen tube protoplast membrane. Membrane currents were recorded upon application of 1000ms of voltage ramped from −200 mV to 0 mV for 50ms, repeated every 5 s.

### Measurements of Ca^2+^ fluxes with non-invasive micromeasurement technology (NMT)

The procedure was done according to a previous report (Michard *et al.*, 2011). Tobacco pollen was germinated in GM for 3h. Pollen tubes 300 μm in length were selected for Ca^2+^ flux measurement. Net fluxes of Ca^2+^ were measured non-invasively using an NMT system BIO-IM (Younger USA, LLC, Amherst, MA, USA). The concentration gradients of the target ions were measured by moving the ion-selective microelectrode between two positions close to the plant material in a pre-set excursion (10 μm for tobacco pollen tubes in the present experiment) at a programmable frequency in the range of 0.3–0.5 Hz. The NMT can measure ionic fluxes down to picomolar levels, but must be measured slowly at ~1–2 s per point.

Pre-pulled and silanized glass micropipettes (2–4 μm aperture, XYPG120-2; Beijing Xuyue Sci. and Tech. Co., Ltd) were first filled with a backfilling solution (Ca^2+^: 100mM CaCl_2_, pH 7.0) to a length of ~1cm from the tip. Then the micropipettes were front filled with ~15 μm columns of selective calcium ionophore I–cocktail A (Sigma 21048, Sigma-Aldrich, St Louis, MO, USA). An Ag/AgCl wire electrode holder (XYEH01-1; Beijing Xuyue Sci. and Tech. Co., Ltd) was inserted in the back of the electrode to make electrical contact with the electrolyte solution. DRIREF-2 (World Precision Instruments) was used as the reference electrode. Ion-selective electrodes of Ca^2+^ and K^+^ were calibrated prior to flux measurements. The calibration media for Ca^2+^ were: 10 μM CaCl_2_ (100 μM KCl, 1.6mM H_3_BO_4_, 50 μM MES, 1% sucrose, pH 5.8); and 100 μM CaCl_2_ (100 μM KCl, 1.6mM H_3_BO_4_, 50 μM MES, 1% sucrose, pH 5.8). Calibration solution for K^+^ are 50 μM KCl (50 μM CaCl_2_, 1.6 mM H_3_BO**_4_**, 50 μM MES, 1 % sucrose, pH 5.8); and 500 μM KCl (50 μM CaCl_2_, 1.6 mM H_3_BO_4_, 50 μM MES, 1% sucrose, pH 5.8). The detailed calibration method is as follows: the electrode was put into germination medium and calibration solution of Ca^2+^, K^+^, respectively, to obtain the voltage number, and then to carry out a linear fit for the voltage and the logarithm of the concentration to obtain the slope and intercept. Only electrodes with Nernstian slopes >26 mV per decade were used in this study. Ion flux was calculated by Fick’s law of diffusion: *J*= –*D* (d*c*/d*x*), where *J* represents the ion flux in the *x* direction, d*c*/d*x* is the ion concentration gradient, and *D* is the ion diffusion constant in a particular medium. Data and image acquisition, preliminary processing, control of the three-dimensional electrode positioner, and stepper motor-controlled fine focus of the microscope stage were performed with Mageflux online software (www.xuyue.net).

Additional methods are provided as supplementary information available at *JXB* online.

### Statistical analysis

The data are expressed as the mean ±SE. Experiments are conducted by the RBD method with three replicates. *P-*values were determined by a one-way analysis of variance (ANOVA) combined with post-hoc analysis in the SPSS 20.0 software.

## Results

### Exogenous GABA modulates tobacco pollen tube growth *in vitro*

**Fig. 1.**
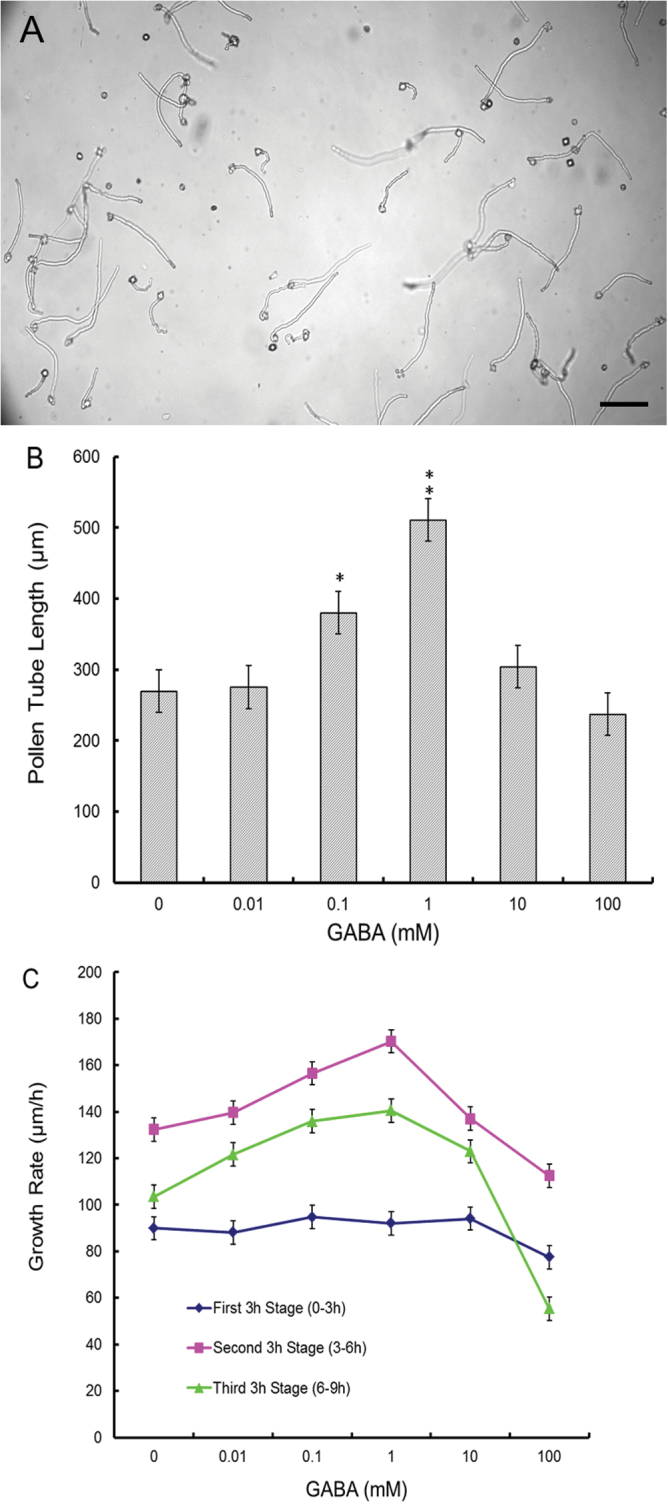
Effect of GABA on *in vitro* cultivated pollen tube growth. (A) Pollen tubes cultured in germination medium for 1h. Scale bar=60 μm. (B) Concentration-dependent effect of GABA on pollen tube growth after 6h culture. (C) GABA dose-dependent growth rate assay. Images of growing pollen tubes were taken via a CCD-coupled microscope at 3h intervals, and then the length of at least 120 pollen tubes was measured with Metamorph software. The average growth rate was calculated every 3h. Means ±SE represent three independent experiments. Significant differences were determined by a one-way ANOVA function combined with post-hoc analysis in SPSS 20.0 software (*P<0.05; **P< 0.01).

To assess the effect of exogenous GABA on tobacco pollen germination and pollen tube growth, pollen grains were germinated in culture media containing different concentrations of GABA. In the GM, pollen tubes grew rapidly and reached 120 μm in length within 1h (Fig. 1A). Significant differences in pollen tube length were observed in the presence of 0.1mM GABA (*P*=0.038) and 1mM GABA (*P*=0.006) after cultivation for 6h (Fig. 1B; Supplementary Fig. S1 at *JXB* online). The average growth rate of pollen tubes treated with 1.0mM GABA was higher than that of pollen tubes treated with GABA at other concentrations (*P*=0.006) (Fig. 1C). However, higher concentrations of GABA (≥10mM) inhibited pollen tube growth as early as the second 3h of cultivation. These results indicate that exogenous GABA stimulates pollen tube growth at lower concentrations, but inhibits pollen tube growth at higher concentrations.

To check whether GABA has a general role in pollen tube growth, pollen of lily (*Lilium concolor*), another widely used model plant, was used to perform a similar experiment. The results indicate that the average length of the GABA-treated pollen tubes (*n*≥40) is much longer than that of the control (without GABA treatment) after 6, 24, and 26h culture (Supplementary Fig. S2 at *JXB* online). These results suggest that GABA is a common signal to regulate pollen tube growth in multiple species with remote evolutionary relationships.

### A GABA gradient exists in tobacco pistils from the stigma to the ovary

**Fig. 2.**
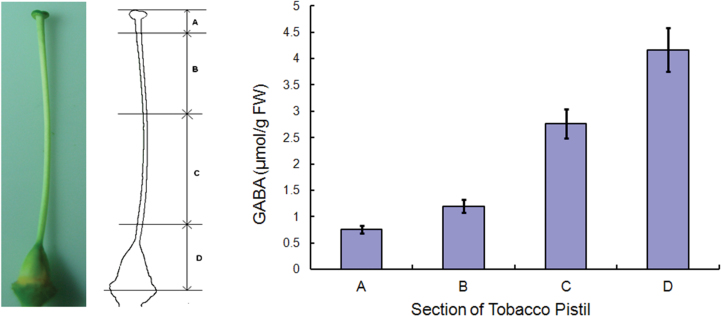
A GABA gradient is present along the length of the pistil. (A–D) Sections of tobacco pistil used for analysis. (A) Uppermost section of the pistil including the stigma; (B) upper section of the style; (C) bottom section of the style; (D) bottom section of the pistil including the ovary. The GABA concentration in each section was determined using an L-8800 automatic amino acid analyser. Means ±SE represents three independent repeats. (This figure is available in colour at *JXB* online.)

A previous investigation showed that *Arabidopsis* pistils exhibit a gradient of GABA levels that increases from the stigma to the ovule micropyle (Palanivelu *et al.*, 2003). To assay whether a GABA gradient exists in tobacco pistils as in *Arabidopsis* pistils, and to obtain evidence for the *in vivo* relevance of exogenous GABA and its effect on pollen tubes demonstrated in this study, physiological concentrations of GABA were further evaluated in tobacco pistils. Tobacco pistils were separated into four sections from stigma to ovary, total free amino acids were extracted from each section, and GABA content was measured using an amino acid analyser (Fig. 2). In this analysis system, the retention time of the derivatized GABA was 23min in the 570nm channel (Supplementary Fig. S3 at *JXB* online). The GABA concentration in the uppermost section (Fig. 2, Section A) was ~0.75 μmol g^–1^ FW (fresh weight). From the top to the bottom of the pistil, the GABA concentration gradually increased ~2-fold in the lower style section and 5-fold in the ovary section (Fig. 2). The calculations indicate that the physiological concentration of GABA in the pistil is in the range of 0.75–4.2mM (Supplementary Note 1). These results demonstrate that a GABA gradient is present in the pistil of tobacco, and might function as an extracellular signal in regulating pollen tube growth.

### Exogenous GABA modulates Ca^2+^-permeable channels in the plasma membrane of pollen tube protoplasts

Previous work demonstrated that exogenous GABA binds to the surfaces of cell membranes of protoplasts and triggers fluctuations of cytosolic Ca^2+^ in these cells (Yu *et al.*, 2006). However, the underlying Ca^2+^ mobilization pathways have remained unclear. To understand further how exogenous GABA modulates Ca^2+^ flow in pollen tubes, protoplasts isolated from germinated pollen grains with growing tubes were used to conduct whole-cell patch-clamp experiments to examine the effects of external GABA on dynamic Ca^2+^ currents. To ensure that the recorded currents were indeed Ca^2+^ currents, the constituents of the bathing and pipette solutions were adjusted in such a way that only Ca^2+^ and Cl^–^ could transit the plasma membrane. The reversal potential of –2.1±0.9 mV (*n*=18) was close to the equilibrium potential for Ca^2+^ (E Ca^2+^=31.6 mV) and far from the Cl^–^ equilibrium potential (E Cl^–^= –156.5 mV), suggesting that the hyperpolarization-activated currents are predominantly caused by Ca^2+^ influx.

**Fig. 3.**
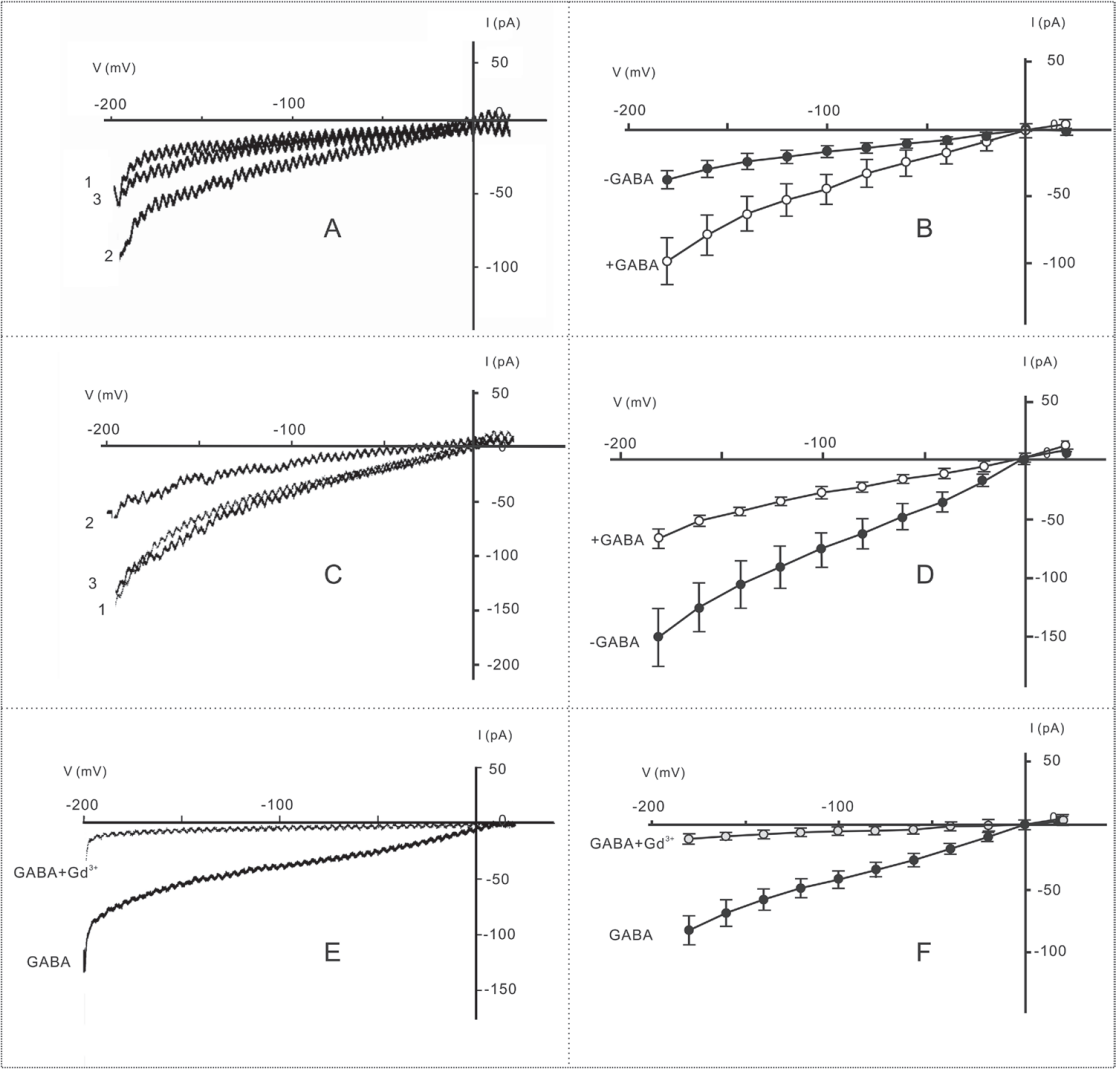
GABA influenced hyperpolarization-activated whole-cell Ca^2+^ currents in tobacco pollen tube protoplasts. (A) Current fluctuation in response to 1.0mM GABA: 1, before GABA addition; 2, treated with 1.0mM GABA; 3, washed with bath solution until the current was stable (*n*=6). (B) Current–voltage relationship of cells treated with 1.0mM GABA (+GABA, *n*=4–6; –GABA, *n*=4–6). (C) Pollen tube protoplasts were treated with 100mM GABA: 1, before GABA addition; 2, treated with 100mM GABA; 3, washed with bathing solution until the current was stable (*n*=6). (D) Current–voltage relationship of cells treated with 100mM GABA (+GABA, *n*=4–6; –GABA, *n*=4–6). (E) Currents induced by 1.0mM GABA before and after addition of 100 μM Gd^3+^ to the bathing solution. (F) Current–voltage relationship of protoplasts before and after adding 100 μM Gd^3+^ to the bathing solution (*n*=5).

The whole-cell Ca^2+^ currents of pollen tube protoplasts are shown in Fig. 3. The membrane potential was ramped from –200 mV to 0 mV within 1 s. When 1.0mM GABA was added to the bathing solution, the Ca^2+^ currents increased within 2min (Fig. 3A), indicating that 1.0mM GABA is sufficient to activate Ca^2+^-permeable channels in the plasma membrane. On average, the Ca^2+^ currents induced by 1.0mM GABA increased from 36.9±7.2 pA to 97.9±18.1 pA at –180 mV (Fig. 3B). Upon washout, the inward current returned to pre-stimulation levels, and this stimulation and restoration pattern could be repeated by adding or removing (by washout) GABA from the bathing solution. This confirmed that Ca^2+^ current alternations were indeed induced by GABA.

Next, Ca^2+^ currents were tested in response to different GABA concentrations. It was found that 0.1mM GABA had no effect on Ca^2+^ currents (*n*=5; Supplementary Fig. S4 at *JXB* online). The 1.0mM GABA treatment, which elicited the maximal stimulatory effect on pollen tube length and growth rate, also significantly enhanced the Ca^2+^ currents (*n*=6; Supplementary Fig. S4). Although the Ca^2+^ currents were observed with 10mM GABA, the extent of the enhancement of Ca^2+^ currents was lower than that observed with 1.0mM GABA. Notably, when 100mM GABA was applied, a concentration that inhibited pollen tube growth, the Ca^2+^ currents were also obviously inhibited (*n*=6; Fig. 3C; Supplementary Fig. S4). On average, compared with the level observed with 1.0mM GABA, currents at –180 mV were inhibited from 153.0±24.8 pA to 68.7±8.7 pA in response to 100mM GABA (Fig. 3D). These results suggest that the enhancement and inhibition of Ca^2+^ currents in response to GABA were consistent with the stimulation and inhibition of pollen tube growth in response to GABA.

To confirm the specificity of GABA-induced Ca^2+^ currents, control experiments were performed by adding gadolinium (Gd^3+^), which specifically blocks Ca^2+^ channels, into the bathing solution. The results showed that GABA-induced currents were significantly inhibited after addition of 100 μM Gd^3+^ (Fig. 3E). On average, the currents at –180 mV were inhibited from 104.5±11.8 pA to 19.58±4.32 pA by Gd^3+^ (Fig. 3F). These results confirm that GABA indeed induces Ca^2+^ current alterations in pollen protoplasts. In addition, these results show that GABA produces biphasic effects on Ca^2+^ currents, activating them at low concentrations and inhibiting them at high concentrations by modulating Ca^2+^-permeable channels in the plasma membrane of pollen tube protoplasts.

### Exogenous GABA increases Ca^2+^ influx in tobacco pollen tubes

To assay dynamic Ca^2+^ flux in growing pollen tubes in response to exogenous GABA, Ca^2+^ influx was measured by NMT (Chen *et al.*, 2009; Michard *et al.*, 2011). This NMT method is based on Fick’s law of diffusion and measures ion flux using specific liquid ion exchanger (LIX) electrodes. To detect Ca^2+^ flux across the cell membrane of pollen tubes in response to exogenous GABA, an electrode containing 10 μM calcium ionophore I (Sigma-Aldrich) was used in stretched micropipettes (Supplementary Fig. S5A at *JXB* online). The results demonstrated that exogenous GABA can indeed trigger the influx of Ca^2+^ in growing pollen tubes; however, the Ca^2+^ oscillation pattern differed in response to different GABA concentrations, and significant oscillation was noticeable with 1.0mM GABA (Supplementary Fig. S5B).

**Fig. 4.**
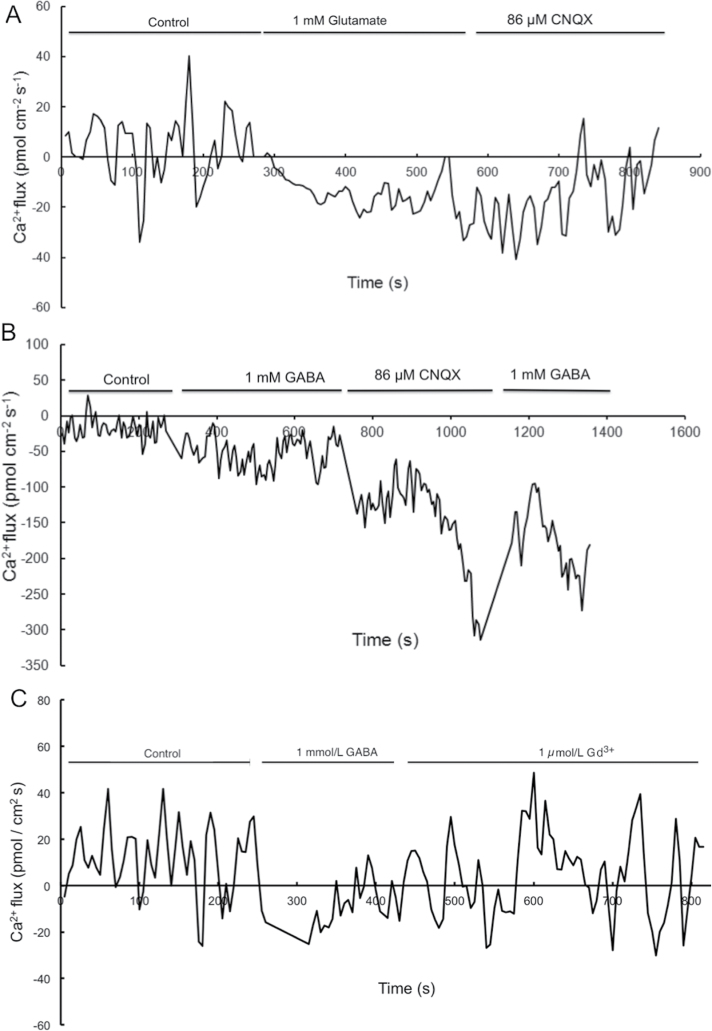
Different patterns of Ca^2+^ flux in response to GABA and glutamate. Data from three experimental blocks were best fit using regression of the moving average model. (A) Ca^2+^ oscillations affected by treatment with 1.0mM glutamate and then with 86 μM CNQX; (B) Ca^2+^ flux recorded at the pollen tube treated first with 1.0mM GABA, then with 86 μM CNQX, and further with 1.0mM GABA; (C) Ca^2+^ oscillations affected by treatment first with 1.0mM GABA and then with 1.0 μM Gd^3+^.

To clarify the specific role of GABA in triggering Ca^2+^ influx, the Ca^2+^ oscillation patterns in pollen tubes exposed to GABA were compared with that in pollen tubes treated with glutamate, the precursor of GABA biosynthesis. The results indicated that 1.0mM glutamate could also trigger the influx of Ca^2+^. However, this influx could be reversed or inhibited by 6-cyano-nitroquinoxaline-2, 3-dione (CNQX) (at 86 μM), a specific inhibitor of one group of ionotropic glutamate receptors (Fig. 4A). This result suggests that tobacco pollen tubes probably express at least one kind of glutamate receptor. However, the oscillation amplitude triggered by 1.0mM GABA was larger than that triggered by glutamate and this was not affected by the addition of CNQX (Fig. 4B). This implied that Ca^2+^ influx caused by GABA is independent of that caused by glutamate. Interestingly, subsequent addition of 1.0mM GABA further triggered Ca^2+^ influx again (Fig. 4B), indicating that GABA-triggered Ca^2+^ influx was not inhibited by CNQX. These data demonstrate that the Ca^2+^ oscillation pattern induced by 1.0mM GABA is different from that triggered by 1.0mM glutamate.

To confirm that Ca^2+^ influx triggered by GABA occurs via Ca^2+^-permeable channels, Gd^3+^, a specific inhibitor of Ca^2+^-permeable channels, was used and Ca^2+^ current influx through pollen tubes was monitored. The results showed that the inward current of Ca^2+^ was markedly reversed by the application of Gd^3+^ at 1.0 μM (Fig. 4C). Thus, the results from both whole-cell patch-clamp and NMT experiments indicate that the inward current of Ca^2+^ induced by 1.0mM GABA is linked to the activation of Ca^2+^-permeable channels in tobacco pollen tube protoplasts and growing pollen tubes.

### GAD is expressed in mature pollen and pollen tubes and co-localizes with GABA at the pollen tube tip

**Fig. 5.**
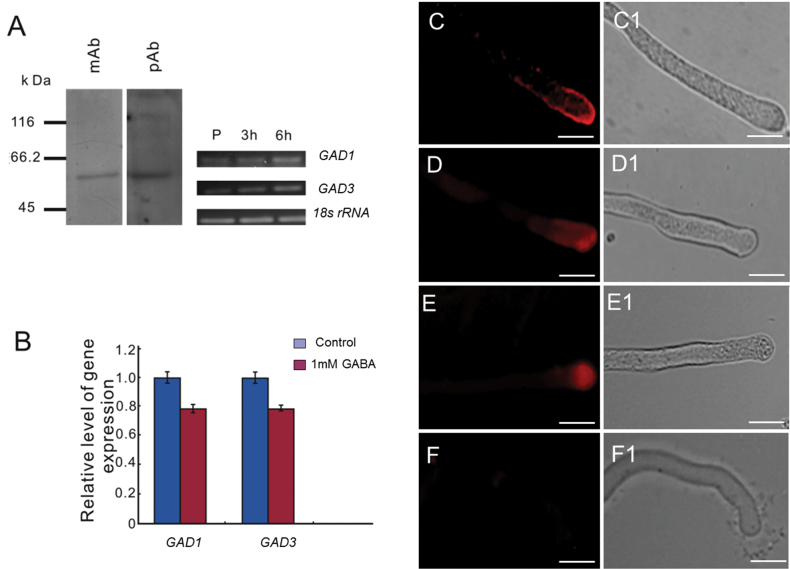
GAD expression analysis in pollen and pollen tubes. (A) Left panel: western blot analysis of GAD protein in tobacco pollen grain extracts. mAb and pAb indicate monoclonal and polyclonal GAD antibodies, respectively; Right panel: RT-PCR detection of *GAD* genes in pollen and pollen tubes. P indicates pollen grains, and 3h and 6h indicate the times of *in vitro* cultivation in germination medium. The *18S rRNA* gene was used as the internal control. (B) The RT-qPCR assay for expression of *GAD* genes in response to treatment with 1.0mM GABA after 6h culture. Means ±SE represent three independent biological repeats. Pollen tubes without GABA treatment and cultured in the same conditions for the same time were used as controls. (C) Fluorescent signal showing localization of GAD (antibody: mAb-GAD 107.1). (D) Fluorescent signal showing localization of CaM (antibody: mAb CaM72-17.28); (E) Fluorescent signal showing localization of GABA. (F) As a control, tobacco pollen tubes were first incubated with immunodepleted antibody and then with TRITC-conjugated secondary antibody in B, C, D. Light microscopy views of the same images are shown in C1–F1. Scale bar=100 μm.

As pollen tubes can grow in the absence of exogenous GABA, it was next questioned how intracellular GAD plays a balancing role in responding to exogenous GABA. GAD is a key rate-limiting enzyme in the GABA biosynthetic pathway and has a domain that interacts with CaM (Baum *et al.*, 1996). Therefore, it was hypothesized that GAD might be a downstream effector of Ca^2+^/CaM to modulate GABA’s effect on pollen tube growth. To test whether GAD accumulates in pollen, total proteins of germinated tobacco pollen were extracted and examined by immunoblotting with either monoclonal (mAb) or polyclonal (pAb) antibodies raised against tobacco GADs (mAb GAD-107.1 and pAb-GAD). mAb GAD-107.1 detected a major band with a mol. wt of ~58kDa, consistent with the expected size of GAD (Chen *et al.*, 1994). In addition, an ~58kDa band was also detected using pAb-GAD (Fig. 5A, left panel). These results indicate that GADs are indeed expressed in tobacco pollen. Real-time PCR (RT-PCR) results provided further evidence that *GAD1* (NCBI Genbank accession no. AF352732) and *GAD3* (accession no. AF353615) are expressed in pollen and pollen tubes; other tobacco *GAD* isoform gene were not detected in pollen tubes. In comparison with the internal control of *18S rRNA* expression, the expression of *GAD* genes was enhanced during pollen tube growth (Fig. 5A, right panel). Quantitative RT-PCR (RT-qPCR) showed that the expression of *GAD* genes in the growing pollen tubes was inhibited by the application of exogenous GABA, suggesting a negative feedback regulation between GABA levels and expression of *GAD* genes (Fig. 5B).

Immunolocalization was carried out to assess the spatial distribution of GAD in pollen tubes using the mAb against GADs. The immunofluorescence signal was preferentially localized in pollen tube tips (Fig. 5C). When pollen tubes were probed with an anti-CaM mAb (CaM72-17.28) that specifically recognizes the GAD-binding domain in CaM (Baum *et al.*, 1996), the fluorescence was also preferentially localized in pollen tube tips (Fig. 5D). Pollen tubes were also stained with an mouse anti-GABA mAb, and it was found that GABA-related fluorescence is primarily found in the pollen tip region (Fig. 5E). No fluorescent signal was detected in control experiments when pollen tubes were treated with immunodepleted antibody plus the secondary antibody (Fig. 5F). These results show that in pollen tubes, GADs and their catalytic product GABA are primarily localized in pollen tube tips, suggesting a regulatory role for GAD/GABA in pollen tube tip growth.

### Pollen tube growth and tip morphology can be altered by a specific inhibitor of GAD

**Fig. 6.**
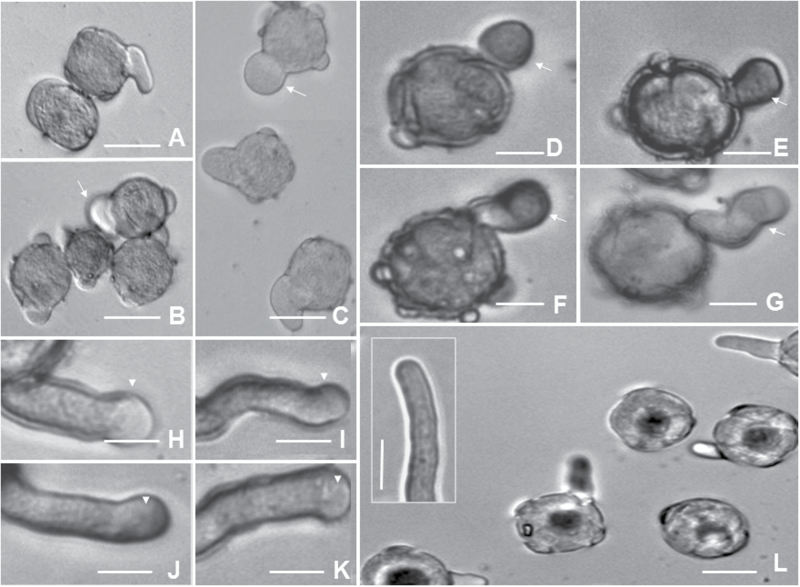
Higher concentrations of 3-MPA altered the morphogenesis of pollen tubes. (A–F) Pollen tubes were treated with 1.0mM 3-MPA. White arrows indicate bulbous pollen tube tips. Scale bar=25 μm. (G–K) Pollen was pre-germinated for 30min and then treated with 1.0mM 3-MPA. Swollen protrusions at the tips of pollen tubes are indicated by arrows. Scale bar=50 μm. (L) Normal pollen tubes in the absence of 3-MPA. Scale bar=50 μm. The insert is a 1/5 magnified image of the pollen tubes. Scale bar=25 μm.

To find evidence of GAD’s involvement in pollen tube growth, 3-MPA, an inhibitor of GAD in neurons (Netopilova *et al.*, 1997), was first evaluated for its role in specific inhibition of plant GAD (Supplementary Fig. S6, Supplementary Note 2 at *JXB* online). 3-MPA was next used in an *in vitro* pollen germination assay to examine the role of GAD in pollen germination and tube growth. At concentrations of 2.0mM and 5.0mM, 3-MPA completely inhibited pollen germination. At 1.0mM 3-MPA, the pollen grains appeared to initiate germination through multiple pores, even though the tube eventually emerged only from one pore. Furthermore, 1.0mM 3-MPA altered the morphology and behaviour of the pollen tubes, which expanded or swelled, and grew in a ‘zigzag’ pattern (Fig. 6A–K). About 68% of the pollen tubes had bulged tips and 24% had broadened shanks even at the first 3h cultivation stage (*n*=400; Fig. 6A–F; Supplementary Fig. S7).

To test further the effect of 3-MPA on pollen tube growth, pollen grains were pre-cultivated in GM for 30min and then treated with 1.0mM 3-MPA. Over 90% of the pollen tubes had abnormalities, with balloon-like protrusions at the tips (Fig. 6G–K). Although 3-MPA at a lower concentration (50 μM) did not noticeably affect the morphology of the pollen tubes, it significantly slowed down pollen tube growth, leading to shorter pollen tubes. Interestingly, treatment with 50 μM 3-MPA supplemented with 1.0mM of exogenous GABA alleviated the inhibitory effect of 3-MPA (*P*=0.042; Supplementary Fig. S8 at *JXB* online). Together, these results indicate that GAD activity is critical for pollen tube growth.

### 3-MPA disrupts actin organization and vesicle trafficking in pollen tubes

**Fig. 7.**
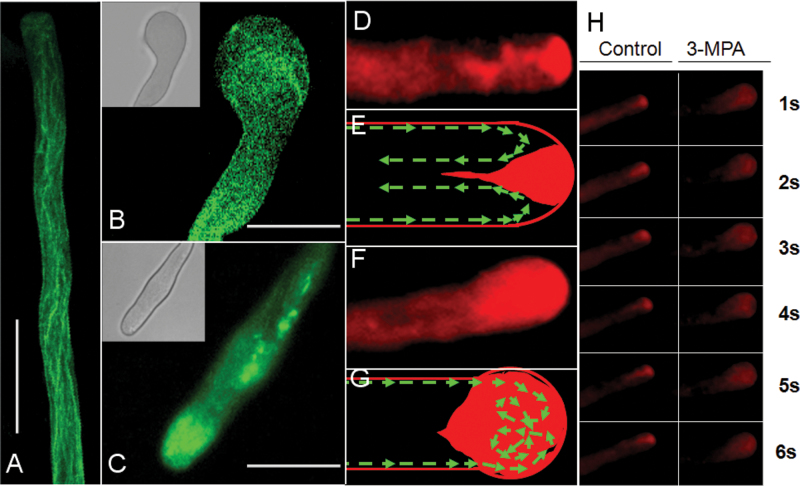
3-MPA altered F-actin organization and vesicle trafficking in pollen tube tips. (A–C) Pollen tubes were treated with 1.0mM 3-MPA at 30min before chemical fixation and FITC–phalloidin staining. (A) Bundles of actin filaments in control cells. (B) Actin filaments in the ballooned tip of a pollen tube. (C) Actin filaments in the broadened shanks of a pollen tube. Inserts show the bright field view of the fluorescence images in about a 1/6 ratio. Scale bar in A=20 μm; scale bar in B, C=40 μm. (D–G) Vesicles were stained with FM4-64, which was internalized within 20min. (D) Control, typical inverse fountain vesicle trafficking (refer to Supplementary Video S1 at *JXB* online). (E) Sketch model of D. The green line and arrowheads indicate the direction of vesicle flow. (F) The direction of vesicle trafficking becomes random after 3-MPA treatment of the balloon-shaped swollen apical pollen tubes (see also Supplementary Video S2). (G) Sketch of vesicle trafficking depicted in F. The dotted line and arrowheads indicate the random direction of vesicle flow. (H) Control and right panels provide observations of vesicle trafficking over time within 6 s without or with 1.0mM 3-MPA treatment, respectively.

To investigate the possible mechanisms of GAD/GABA action in pollen tube growth regulation, abnormally growing pollen tubes treated with 3-MPA were further analysed. As described above, 1.0mM 3-MPA altered the morphology of pollen tubes within 30min, causing either balloon-shaped or swollen tips, and broadened pollen tube shanks (Fig. 7, insets). Actin plays a critical role in the morphogenesis of pollen tubes (Foissner *et al.*, 2002; Cvrckova *et al.*, 2004); therefore, it was investigated whether the morphological alteration observed upon 3-MPA treatment was caused by disruption of actin organization. In untreated samples, longitudinal bundles of F-actin were distributed along the shanks of the pollen tubes and were absent near the pollen tube apex (Fig. 7A). After application of 1.0mM 3-MPA, actin became disorganized in the shanks of the pollen tubes, particularly in the balloon-shaped tip (Fig. 7B), perhaps causing broadening of the pollen tube shank (Fig. 7C). These results imply that GAD is required for maintenance of dynamic actin organization and the normal morphology of pollen tubes.

During pollen tube growth, secretory vesicles are delivered to the tip via actin microfilaments. A highly efficient vesicular transport system, mobilized by the actin cytoskeleton, supports rapid tip growth in pollen tubes (Hepler *et al.*, 2001). To monitor the extent to which vesicle trafficking is affected by 3-MPA, vesicle trafficking was tracked with the vesicle-specific fluorescent dye FM4-64. As expected, in pollen tubes grown without 3-MPA, secretory vesicles exhibited rapid bidirectional cytoplasmic streaming, known as a reverse fountain movement (Fig. 7D–E; H, control panel; Supplementary Video S1 at *JXB* online). However, in the balloon-shaped tips of pollen tubes grown in the presence of 3-MPA, the direction of vesicle trafficking was random (Fig. 7F, G; H, right-hand panel; Supplementary Video S2). These results raise the possibility that actin filament disorganization and misdirected vesicle trafficking caused by 3-MPA-treatment led to the abnormal pollen tube morphology.

### Ca^2+^ efflux caused by 3-MPA can be reversed upon GABA addition

**Fig. 8.**
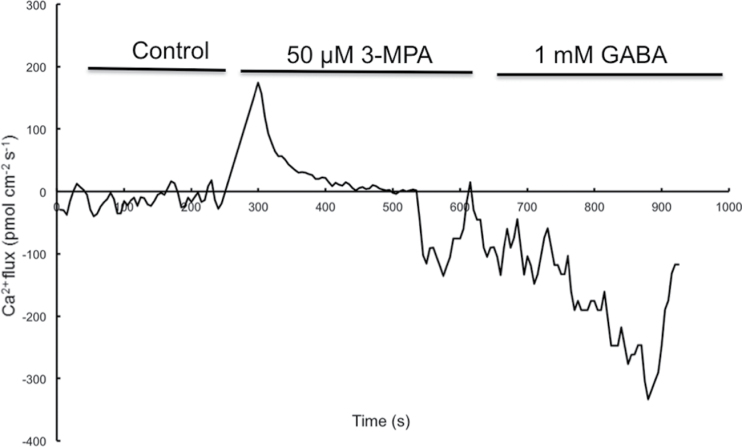
Ca^2+^ oscillations were affected by 3-MPA. Data from three experimental blocks were best fit using a regression of moving average model. Ca^2+^ oscillations affected by 3-MPA and GABA compared with control. First treatment with 50 μM 3-MPA and then with 1.0mM GABA.

Ca^2+^ oscillation is a sensitive and early indicator of extracellular stimulation. Ca^2+^ oscillation was observed when pollen tubes were exposed to 3-MPA. This result showed that addition of 3-MPA at 50 μM triggered an obvious efflux of Ca^2+^ (Fig. 8); however, this Ca^2+^ efflux was noticeably reversed by the addition of 1.0mM GABA (Fig. 8). This result suggests that this re-triggered Ca^2+^ influx might be due to the function of GABA in inducing Ca^2+^ influx at the early time points after the addition of GABA.

### Expression of multiple putative downstream signalling components may be activated in response to GABA

**Fig. 9.**
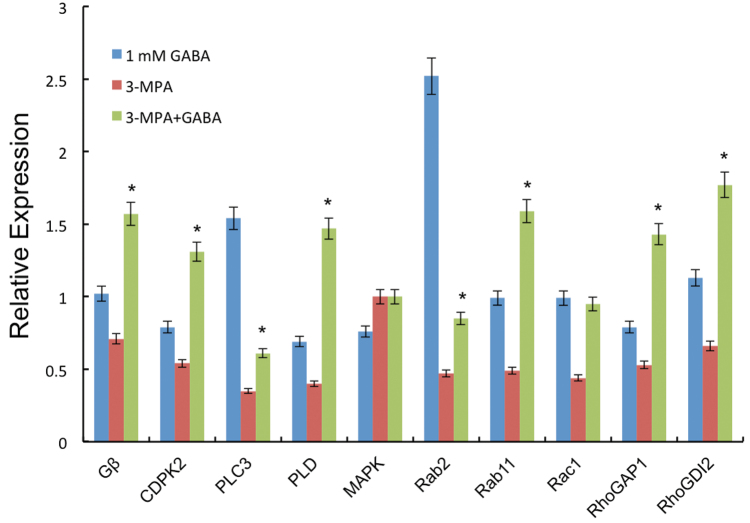
Expression of genes for various signalling molecules in response to different treatments. The transcript level ratio was normalized to the *18S rRNA* gene; the relative expression level was calculated by the ΔΔCT method. The expression levels of *Gβ*, *CDPK2*, *PLC3*, *PLD*, *MAPK*, *Rab2*, *Rab11*, *Rac1*, and *Rho GAP1RhoGDI2* upon GABA (1.0mM) and 3-MPA (50 μM) plus GABA (1.0mM) treatments, respectively. Results represent means ±SE of three independent repeats. Significant differences between different treatments were calculated via a one-way ANOVA combined with post-hoc analysis. *P<0.05. (This figure is available in colour at *JXB* online.)

To screen the possible effectors of GABA signalling in pollen tube growth, RT-qPCR was used to measure the gene expression of multiple regulatory/signal molecules. These components are involved in vesicle trafficking and polarity signal transduction pathways that are crucial for pollen tube tip growth. The heterotrimeric Gβ subunit (Ma *et al.*, 1999) and its possible downstream signalling components PLC3 (Dowd *et al.*, 2006; Helling *et al.*, 2006), PLD (Potocky *et al.*, 2003), MAPK (Wilson *et al.*, 1995), and CDPK2 (calcium-dependent protein kinase) (Yoon *et al.*, 2006) are involved in the regulation of pollen tube growth. Moreover, small G proteins such as Rab2 and Rab11 participate in vesicle trafficking in tobacco pollen tube tip growth (Cheung *et al.*, 2002; de Graaf *et al.*, 2005). Additional regulatory molecules, such as guanine nucleotide dissociation inhibitors (GDIs) and GAP1 (GTPase-activating protein gene), play critical roles in the regulation of molecular switch activities (Cheung *et al.*, 2003; Klahre and Kost, 2006; Klahre *et al.*, 2006). RT-qPCRs showed that the expression of *PLC3* and *Rab2* increased markedly in pollen tubes grown in the presence of exogenous GABA, 0.5- and 1-fold higher than that in the control (the ratio was normalized to the internal housekeeping *18S rRNA* gene), respectively. *RhoGDI2* expression increased by 0.3-fold. After 3-MPA treatment, the expression of multiple genes, excluding *MAPK* which did not show an obvious change, was decreased (Fig. 9). However, compared with 3-MPA treatment, 3-MPA plus GABA markedly increased the expression of *Gβ* (1-fold higher), *CDPK2* (1.5-fold higher), *PLD* (2.2-fold higher), *Rab11* (2-fold higher), *RhoGAP1* (1.5-fold higher), and *RhoGDI2* (1.5-fold higher). In addition, *PLC3*, *Rab2*, and *Rac1* also increased ~0.5- to 1-fold, and *MAPK* appeared not to be affected by GABA treatment (Fig. 9).

## Discussion

In their long journey to the ovules, pollen tubes must penetrate the style and respond properly to various signals from the pistil tissue to ensure their correct arrival at an ovule (Sauter, 2009). Among these signals, GABA from the pistil is a critical guidance clue (Palanivelu *et al.*, 2003). In the present work, it was found that an optimum concentration of exogenous GABA enhanced pollen tube growth, but a high concentration of GABA inhibited growth (Fig. 1). It was further confirmed that a gradient of GABA exists in the tobacco style, and the 1.0mM exogenous GABA applied in this research also falls within the physiological concentration. This result is consistent with previous observation of *Arabidopsis* (Palanivelu *et al.*, 2003). This suggests that variations in GABA concentration in the style regulate the pace of pollen tube growth (Fig. 2). Evidence is also provided for the mechanism by which exogenous GABA regulates pollen tube growth. In this contribution, Evidence is provided to show the possible mechanism of GABA involvement in the enigmatic fertilization courses of plants.

### Pistil–pollen tube communication: exogenous GABA regulates Ca^2+^ influx by modulating Ca^2+^-permeable channels

Although pistils of different species have been reported to contain many factors that play roles in pollen tube growth (Kim *et al.*, 2003; Marton *et al.*, 2005; Okuda *et al.*, 2009), the molecular mechanisms of communication between the pistil and pollen tube remain largely unknown. Previously, it was found that GABA can bind to the membrane surface of *Arabidopsis* and tobacco pollen protoplasts, trigger fluctuations in cytosolic Ca^2+^, and enhance Ca^2+^ levels in the cells (Yu *et al.*, 2006). In the present work, it was confirmed that GABA affects Ca^2+^ influx by modulating Ca^2+^-permeable channels (Fig. 3). The results show that exogenous GABA can affect Ca^2+^ currents in a concentration-dependent biphasic manner in tobacco pollen tube protoplasts and growing pollen tubes, activating the Ca^2+^-permeable channels at lower concentrations and inhibiting the channels at higher concentrations. Previous research confirmed that pollen tubes have the same type of Ca^2+^-permeable channels as pollen grains (Wang *et al.*, 2004). Studies showed that d-serine can regulate Ca^2+^-permeable channels via the activation of a glutamate receptor-like receptor (a type of Ca^2+^ channel) in pollen tubes (Michard *et al.*, 2011). The present results indicate that GABA can also activate Ca^2+^ channels. The results of NMT offer further support that this possible ligand-gated activation of Ca^2+^-permeable channels occurs as a specific response to GABA (Fig. 4). Therefore, it is reasonable to deduce that the effects of GABA on pollen tube growth are mainly transmitted via its modulation of Ca^2+^-permeable channels. This suggests that GABA, as an external cue, mediates communication between the pistil and pollen tubes.

Notably, the relatively high influx of Ca^2+^ was still observed in the presence of CNQX (at 86 μM) and the addition of GABA led to further Ca^2+^ influx (Fig. 4). This indicates that the GABA-triggered Ca^2+^ influx was not inhibited by CNQX and is independent of that triggered by glutamate. CNQX was used as a specific inhibitor of ionotropic glutamate receptors in previous research (Michard *et al.*, 2011). There is a possibility that the blockage of ionotropic glutamate receptors by CNQX might stimulate GABA-triggered Ca^2+^ influx in compensation. Thus, CNQX treatment may have no specific influence on GABA-triggered Ca^2+^ influx as observed in Fig. 4. It is reasonable that multiple Ca^2+^ channels are present on the plasma membrane in responding to various extracellular signals during pollen tube growth.

It was also observed that GABA could trigger the tendency for K^+^ outflux (Supplementary Figs S9, S10 at *JXB* online) in addition to activating Ca^2+^ channels. This is consistent with a previous report showing that the outward K^+^ channel is reciprocally regulated by Ca^2+^ channel activation in *Pyrus pyrifolia* pollen (Wu *et al.*, 2011). Furthermore, K^+^ outward rectifying flux induced by GABA is also different from that caused by glutamate (Supplementary Fig. S10, Supplementary Note 3). These results imply that the activation of Ca^2+^ and K^+^ channels might be simultaneously regulated by the binding of GABA to the putative receptor, as occurs in animal cells (Blein *et al.*, 2000; Niswender and Conn, 2010). These data substantiate the possibility that proteins analogous to the metabotropic GABA_B_ receptor exist on the membrane of pollen tubes and provide an important tool to understand the mechanism of GABA-mediated pollen tube–pistil communication.

### GAD/CaM probably acts as a rheostat of Ca^2+^ to integrate its downstream signalling

GAD has an autoinhibitory domain in its C-terminal region and can bind to a Ca^2+^/CaM complex. When cytosolic Ca^2+^ increases, Ca^2+^/CaM binds to GAD, resulting in the rapid induction of GABA synthesis (Chen *et al.*, 1994). It was previously shown that exogenous GABA can bind to the cell membrane, resulting in oscillation of Ca^2+^ in tobacco pollen, and indicating that exogenous GABA can affect the levels and distribution of Ca^2+^ in cells (Yu *et al.*, 2006). In the present work, it was confirmed that GAD, GABA, and CaM are closely co-localized in the growing tips of pollen tubes (Fig. 5). It was also demonstrated that GAD is mainly localized on the membrane at the pollen tube tips. This result is consistent with the previous prediction that GAD is associated with the cell membrane (Alexandersson *et al.*, 2004). Interestingly, it was revealed that CaM could probably form a complex with Ca^2+^ channels to sense the Ca^2+^ source (Tadross *et al.*, 2008). This suggests that GAD and CaM may be directly associated with Ca^2+^ channels and function as a complex. It is possible that exogenous GABA modulates Ca^2+^-permeable channels by activating G protein-coupled receptors (GPCRs) and regulating GAD activity via CaM, which is associated with a Ca^2+^-permeable channel. To test this idea, a GAD inhibitor, 3-MPA, was applied which led to significantly slowed pollen tube growth and shorter pollen tubes with swollen and twisted tips (Figs 6, 7). The inhibition of pollen tube growth by 3-MPA treatment could be partially alleviated by exogenous GABA. Proteomic and cytological analyses have revealed the important role of the Ca^2+^/CaM complex in pollen tube growth (Chen *et al.*, 2009). Although the complicated interlink of actin organization, vesicular trafficking, and Ca^2+^ dynamics made it difficult to distinguish the cause and the consequence among them in relation to altered morphology after inhibitor treatment, the present data clearly show that GAD seems to be a key component of the Ca^2+^/CaM downstream targets and plays a critical role in the regulation of pollen tube growth as a downstream modulator of Ca^2+^/CaM in response to exogenous GABA.

### Multiple signal pathways may be involved in perceiving exogenous GABA

The mechanisms of GABA function in pollen tube growth, although interesting and important (Ma, 2003; Palanivelu *et al.*, 2003; Yang, 2003), remain largely unknown. The present data revealed that the inhibition of GAD resulted in abnormal actin organization and vesicle trafficking, and thus abnormal pollen tube growth. To understand its relationship to known signalling pathways involved in pollen tube growth, some key components involved in signal transduction and vesicle trafficking in pollen tube growth were tested. Previous research demonstrated that PLC3, PLD, and CDPK are all involved in plasma membrane polarization and polar pollen tube growth (Potocky *et al.*, 2003; Dowd *et al.*, 2006; Helling *et al.*, 2006). The present RT-qPCR results indicated that exogenous GABA significantly stimulates the expression of *PLC3* and *Rab2* during normal pollen tube growth (Fig. 9). However, when endogenous GABA synthesis was inhibited by 3-MPA, the expression of these genes was reduced, and exogenous GABA only markedly increased the expression of *Gβ*, *CDPK2*, *PLD*, *Rab11*, *RhoGAP1*, and *Rho GDI2*. Although the activities of these signalling effectors were not assayed at the protein level, the present data suggest that there might be at least three common signalling pathways, PLC3, PLD, and CDPK2, that respond to GABA. Moreover, in the interlinked signal pathways, GAD and its regulator CaM may act as a Ca^2+^ rheostat to maintain the balance of Ca^2+^ and modulate the GABA level in pollen tubes by the feedback modulation on the Ca^2+^ channel. Although exogenous and endogenous GABA signals may trigger the activation of different pathways, they finally converge to regulate F-actin and vesicle trafficking directly or indirectly, as well as regulating subsequent pollen tube tip growth (Dowd *et al.*, 2006).

In summary, the flux of ions across membranes via ion channel activation is vital to cellular responses to internal and external stimuli, and therefore crucial for cellular survival in changing circumstances. It was previously reported that exogenous GABA can trigger an increase in cytosolic Ca^2+^ in tobacco pollen protoplast and it was proposed that Ca^2+^ influx occurs across the plasma membrane. However, the upstream stimuli that activate plasma membrane-localized Ca^2+^ channels and downstream signalling transduction pathway were unknown. Here evidence is provided for the activation of Ca^2+^-permeable channels coupling outward K^+^ efflux in the plasma membrane of tobacco pollen tubes in response to exogenous GABA. When pollen tubes pass through a GABA gradient in the tobacco style, this extracellular GABA might efficiently modulate pollen tube growth in a dose-dependent manner by modulating Ca^2+^-permeable channels on the pollen tube membrane, triggering Ca^2+^ influx in pollen tubes, and thus mediating GAD/CaM signalling. In addition, the Ca^2+^ flux pattern triggered by GABA is different from that induced by glutamate, indicating its specificity in activating Ca^2+^-permeable channels. In brief, GABA as a signalling molecule mediates style–pollen tube communication and regulates pollen tube growth though Ca^2+^ signalling.

## Supplementary data

Supplementary data are available at JXB online.

Supplementary methods

Figure S1. The impact of different GABA concentrations on tobacco pollen tube growth. Photographs were taken at 6h cultivation

Figure S2. Effect of GABA on lily pollen tube growth.

Figure S3. Content of amino acids analysed by an L-8800 automatic amino acid analyser.

Figure S4. Fold change of whole-cell currents at –200 mV against different GABA concentrations. *n*=4–6.

Figure S5. Ca^2+^ oscillations recorded by non-invasive micromeasurement technology (NMT).

Figure S6. Specific inhibition by 3-MPA of the production of GABA.

Figure S7. Percentage of the different abnormal pollen tubes after the application of 1.0mM 3-MPA for 3h.

Figure S8. The growth inhibition by a low concentration of 3-MPA was ameliorated by addition of exogenous GABA.

Figure S9. K^+^ oscillation pattern responding to different GABA concentrations.

Figure S10. Influence of CNQX after GABA (A) and glutamate (B) treatment on K^+^ flux in the tip of tobacco pollen tubes.

Table S1. Gene-specific primers used in RT-qPCR assay.

Video S1. Time-lapse video showing the vesicle trafficking pattern in normal pollen tubes.

Video S2. Time-lapse video showing the vesicle trafficking pattern in pollen tubes after 1.0mM 3-MPA treatment.

Note 1. Calculation of GABA concentration in tobacco style.

Note 2. Specific inhibition by 3-MPA of GAD activity.

Note S3. K^+^ flux responds to GABA.

Supplementary References

Supplementary Data

## Abbreviations:

CaM: calmodulin
CNQX: 6-cyano-nitroquinoxaline-2, 3-dione
GABA: γ-aminobutyric acid
GAD: glutamate decarboxylase
3-MPA: 3-mercaptopropionic acid
TRITC: tetramethylrhodamine-5-(and 6)-isothiocyanate.
