# Supplementary material for: Exogenous γ-aminobutyric acid (GABA) affects pollen tube growth via modulating putative Ca2+-permeable membrane channels and is coupled to negative regulation on glutamate decarboxylase
Source: J Exp Bot. 2014 May 5;65(12):3235–48. doi: 10.1093/jxb/eru171 (PMC4071839; doi:10.1093/jxb/eru171)
Supplement: Supplementary Data [file supp_65_12_3235__index.html]

Exogenous γ-aminobutyric acid (GABA) affects pollen tube growth via modulating putative Ca2+-permeable membrane channels and is coupled to negative regulation on glutamate decarboxylase — Supplementary Data 

# Exogenous γ-aminobutyric acid (GABA) affects pollen tube growth via modulating putative Ca2+-permeable membrane channels and is coupled to negative regulation on glutamate decarboxylase

## Supplementary Data

Data files

**Files in this Data Supplement:**

- Supplementary Data - Supplementary Data
- Supplementary Data - Supplementary Data
- Supplementary Data - Supplementary Data
