# Supplementary material for: Exogenous γ-aminobutyric acid (GABA) affects pollen tube growth via modulating putative Ca2+-permeable membrane channels and is coupled to negative regulation on glutamate decarboxylase
Source: J Exp Bot. 2014 May 5;65(12):3235–48. doi: 10.1093/jxb/eru171 (PMC4071839; doi:10.1093/jxb/eru171)
Supplement: Supplementary Data [file supp_eru171_JEB_Supporting_data_Correction.doc]

**Supplementary Information**

**Exogenous GABA affects pollen tube growth via modulating putative Ca^2+^-permeable membrane channels and is coupled with negative regulation on glutamate decarboxylase**

Guang-Hui Yu, Jie Zou, Jing Feng, Xiong-Bo Peng, Ju-You Wu, Ying-Liang Wu, Ravishankar Palanivelu, Meng-Xiang Sun

Supplementary data includes additional methods，10 figures, 1 table, 2 videos and 3 Notes and references.

Supplementary Methods

Supplementary Figures

Figure S1. The impact of different GABA concentrations on tobacco pollen tube growth. Photographs taken at 6 h cultivation

Figure S2. Effect of GABA on lily pollen tube growth.

Figure S3. Content of amino acids was analyzed by L-8800 automatic amino acid analyzer.

Figure S4. Fold change of whole-cell currents at -200 mV against different GABA concentrations. N=4-6.

Figure S5. Ca^2+^ oscillations recorded by Non-invasive Micro-test Technology (NMT).

Figure S6. Specific inhibition of 3-MPA on the production of GABA.

Figure S7. Percentage of the different abnormal pollen tubes after the application of 1.0 mM 3-MPA application for 3 h.

Figure S8. The growth inhibition by low concentration of 3-MPA was ameliorated by addition of exogenous GABA.

Figure S9. K^+^ oscillation pattern responding to different GABA concentration.

Figure S10. Influence of CNQX after GABA（A）, Glu（B）treatment on K^+^ flux in the tip of tobacco pollen tubes.

Table S1. Gene-specific primers used in RT-qPCR assay.

Video S1. Time-lapse video showing vesicles trafficking pattern in normal pollen tubes.

Video S2. Time-lapse video showing vesicle trafficking pattern in pollen tubes after 1.0 mM 3-MPA treatment.

Note S1. Calculation of GABA concentration in tobacco style;

Note S2. Specific inhibition of 3-MPA on GAD activity；

Note S3. K^+^ flux responds to GABA.

Supplementary References

**Supplementary methods**

**Assay of Lily pollen tube growth**

The pollen of Lily (Lilium concolor) were collected from the dehiscence anthers, and germinated on solid modicum containing 50 g/L Sucrose+H3BO3 20 mg/L+CaCl2 20 mg/L with 0.8% agar (1 mM MES, pH=5.8). After cultured for 24 h, the growing pollen tubes were photographed by stereo microscope (Nikon SMZ1500). The micrometer rule was used as the standard criterion to measure the length of the pollen tubes aided by image J software. At least 40 pollen tubes were randomly chosen for measurement at each time point. This measurement was conducted by blind experiment.

**Immunofluorescence microscopy**

To observe the subcellular localization of GABA, GAD, and CaM, tobacco pollen tubes collected after *in vitro* germination for 3–4 h were fixed at room temperature for 2 h in a solution containing 4% paraformaldehyde (v/v), 50 mM PIPES (1,4-piperazinediethanesulfonic acid) buffer, pH 6.9, 2 mM MgSO_4_, and 10% sucrose. After washing with PBS (10 mM phosphate-buffed saline, pH 7.4, 138 mM NaCl, 2.7 mM KCl), the fixed pollen tubes were blocked with 3% (v/v) nonfat dry milk in PBST (PBS containing 0.005% [v/v] Triton X-100) at room temperature for 1 h. The cells were then reacted with anti-GABA monoclonal antibody (50× dilution, Sigma), anti-GAD monoclonal antibody (mAb-GAD 107.1, 50× dilution), or anti-CaM monoclonal antibody (mAb CaM72-17.28，50× dilution), at 30°C for 1–2 h. The monoclonal and polyclonal anti-GAD antibodies（mAb GAD-107.1 and pAb-GAD）and the anti-CaM antibody (mAb CaM72-17.28) were generously provided by Prof. Hillel Fromm (Tel Aviv University, Israel); procedures were conducted according to their previously reports (Baum et al., 1996; Snedden et al., 1996). The anti-CaM monoclonal antibody (mAb CaM72-17.28) also precipitated the tobacco 58 kDa GAD based on previous reported. The primary antibodies were diluted in PBS buffer containing 1% non-fat dry milk. After three 10-min washes in PBST, the slides were incubated with a secondary antibody (TRITC-conjugated goat F(ab)_2_ fragment against mouse IgG (Sigma); 100× dilution in PBS containing 1% (v/v) non-fat milk) at 30°C for 1 h. As a control，tobacco pollen tubes were incubated with immuno-depleted antibody and then with the second antibody. The slides were then washed as described above and mounted in a PBS solution containing 0.1% (v/v) *р*-phenylenediamine and 50% (v/v) glycerol. The samples were observed under an inverted DMIRE2 fluorescence microscope (Leica). Negative controls were included in which the primary antibody was omitted.

**Visualization of pollen tube actin**

To visualize the effect of 3-MPA on F-actin in pollen tubes, after 1 h of cultivation, pollen tubes were cultured in GM supplemented with 1.0 mM 3-MPA for 0.5 h and then fixed. The fixative contained 3.7% formaldehyde, 0.1 mM K-phosphate buffer (pH 7.0), 10 mM EGTA, 5.0 mM MgSO_4_, and 20% sucrose. After fixation for 30 min, the pollen tubes were washed three times for 5 min each with GM, and then FITC-labeled phalloidin (5.0 µM) was added to the GM for 30 min. Unbound dye was then washed out. Fluorescence images were obtained with a confocal microscope (model TCS SP5; Leica). A reconstruction function was employed to process the images.

**FM4-64 staining of pollen tube vesicles**

To visualize the effect of 3-MPA on vesicle trafficking in the pollen tube apices, cells were loaded with FM4-64. The dye (5–10 µM) was added to the germination medium. Time-lapse images were acquired using a Leica DMIRE2 inverted microscope equipped with a cooled CCD (model RTE/CCD-1300-Y/HS, Roper Scientific Co.) and processed with Metamorph software.

**Plant protein extraction, electrophoresis, and protein gel blot analysis**

Isolation total proteins from tobacco pollen are based on previous EZ method (Martinez-Garcia et al., 1999). 100 mg of pollen was frozen in liquid nitrogen and ground to a fine powder with 0.2 mg of quartz sand with a mortar and pestle. The powder was resuspended in 0.3 mL of buffer E (125 mM Tris-HCl, pH 8.8, 1% (w/v) SDS, 10% glycerol, 50 mM Na_2_S_2_O_5_). The solution was centrifuged at 8,000 rpm for 10 min at 4°C. The supernatant was combined with 30 µL of buffer Z (125 mM Tris-HCl, pH 6.8, 12% (w/v) SDS, 10% glycerol, 22% (v/v) β-mercaptoethanol, 0.001% bromophenol blue) and boiled for 10 min. The sample was then centrifuged at 8,000 rpm for 10 min. Then, 25 µL of supernatant containing the protein was loaded on a 10% SDS-polyacrylamide gel, and the proteins were separated by electrophoresis. The proteins were transferred to a Protran nitrocellulose membrane (Schleicher & Schuell, Germany) and reacted with anti-GAD monoclonal antibody (500× dilution, supplied by Prof. Hillel Fromm). The antibody was detected with horse anti-mouse IgG secondary antibody conjugated with alkaline phosphatase (500× dilution, Zhongshan Golden Bridge Biotechnology Co., Ltd.). Membranes soaked in CSPD chemiluminescent substrate solution (100× dilution, Roche Molecular Biochemicals) were exposed to X-ray film for 30 min. The images were scanned using ArtixScan 2500. To confirm equivalent protein loading, the proteins were visualized by staining the membrane with Ponceau S concentrate (Sigma) after transfer. Protein molecular weight markers (Fermentas Life Sciences) were used to estimate protein sizes.

**Measurement of GABA contents**

Free amino acids were extracted from fresh tobacco pistils, pollen grains, and pollen tubes as previously described (Liu et al., 2011). The extract was centrifuged at 15,000 rpm for 10 min followed by filtration of the supernatant with a 0.22-μm filter. GABA content analysis was performed with an automatic amino acid analyzer (L-8800; Hitachi High-Technologies, Tokyo, Japan) using post-column derivatization. Briefly, 10 µL of the above extract was injected directly into the analyzer onto a column packed with a custom ion-exchange resin (4.6 × 60 mm, 5-μm particle size; Hitachi 2622), with the temperature held at 57°C. The lithium citrate buffer and ninhydrin flow rates were 0.40 and 0.35 mL/min, respectively. The reaction coil temperature was set to 135°C. Two separate analyses (two detection channels at 570 and 440 nm, respectively) were carried out for each sample. GABA contents of the samples were calculated by comparison with the areas of the amino acid standards.

**Enzyme activity assay**

Total proteins for enzymatic assays were extracted from tobacco pollen grains or pollen tubes as previously described (Chen et al., 1994). The GAD enzymatic assay was performed by directly measuring GABA production after the GAD reaction: 600 µg of total protein was incubated in 200 µL of reaction mixture containing 50 mM sodium phosphate (pH 5.8), 10 mM glutamic acid, and 0.2 mM pyridoxal 5'-phosphate (PLP). Reactions were performed at 30°C for 24 h and terminated by the addition of 200 µL of 200 mM sodium borate (pH 9.0). The GABA content was spectrophotometrically determined by the Berthelot color reaction according to a previous method (Johnson et al., 1997). The color was visualized by adding 1 mL of 6% phenol solution and 400 µL of 5.25% sodium hypochlorite, followed by incubation in boiling water for 10 min and then immersion in an ice bath for 20 min. The content of GABA was determined by measuring the absorption at 630 nm and comparing to a standard curve. GAD activity was defined as the amount of GABA (µmol) produced per µg protein and per hour.

Superoxide dismutase (SOD) activity in gel assay was determined by staining with nitrobluetetrazolium (NBT) after native polyacrylamide gel electrophoresis (Native PAGE) (Wang et al., 2009). The lanes of SOD gels were cut for further treatment with 3-MPA (0.1 mM). As the controls, the gels were treated or not treated with H_2_O_2_ (3% concentration).

**RNA extraction and real-time quantitative PCR**

After pollen (about 50 mg) had germinated in 50 mL of GM (see above) for 3 or 6 h, the medium was centrifuged for 10 min at 12,000 rpm to collect the germinating pollen tubes. The tubes were ground to a fine powder in liquid nitrogen with 0.2 mg of quartz sand in a mortar, and then the powder was transferred to a centrifuge tube pretreated with liquid nitrogen and used for RNA extraction. RNA was extracted using [Bioteke RNApure Rapid Superpure Total RNA Extraction Kit](http://www1.qiagen.com/Products/RnaStabilizationPurification/RNeasySystem/RNeasyPlantMini.aspx) (Bioteke Technology Corporation, Beijing). Then, cDNA was synthesized from 0.5 μg of total RNA using the ReverTra Ace-α- kit (Toyobo, Shanghai) according to the manufacturer's instructions. The RNA concentrations were determined by UV spectrophotometry (Biomate 5, Thermo Spectronic). The same amount of cDNA was used for RT-qPCR of samples from the different treatments.

RT-qPCR was performed in a Line-Gene K Sequence Detection System (Hangzhou Bioer Technology Co., China). Briefly, 20 µL of reaction mixture containing 0.5 µL of cDNA template (50× dilution of cDNA), 1.0 µL each of forward and reverse primers (10 μM), 10 µL of 2× SYBR real-time PCR premixture (BioTeke Technology Corporation, Beijing), and 7.5 µL ddH_2_O were amplified as follows: denaturation at 95°C for 3 min and 35 cycles at 95°C for 30 s, various annealing temperatures (see Table S1 in Supplementary information) and 72°C for 30 s. The different temperatures used for each pair of primers are shown in Table S1. RT-qPCR reactions were performed in triplicate for each sample, and the mean value was used to calculate the mRNA levels. Quantitative analysis was performed using the comparative CT method. The mRNA copy numbers of target genes in pollen were normalized to the mRNA copy numbers of the housekeeping gene *18s rRNA* to give a value for ΔCT. The transcriptional levels of the target genes in different treatments were then normalized to their corresponding control samples giving ΔΔCT; the relative expression of target signal genes in the treated samples was expressed as 2^−ΔΔCT^. This final step was to determine the gene expression level with each treatment. *18S rRNA* expression was used as an internal control. The forward and reverse primers used for quantification are listed in Table S1. At the end of each PCR run, data were automatically analyzed by the system and ampliﬁcation plots were obtained.

**Supplementary Figures**


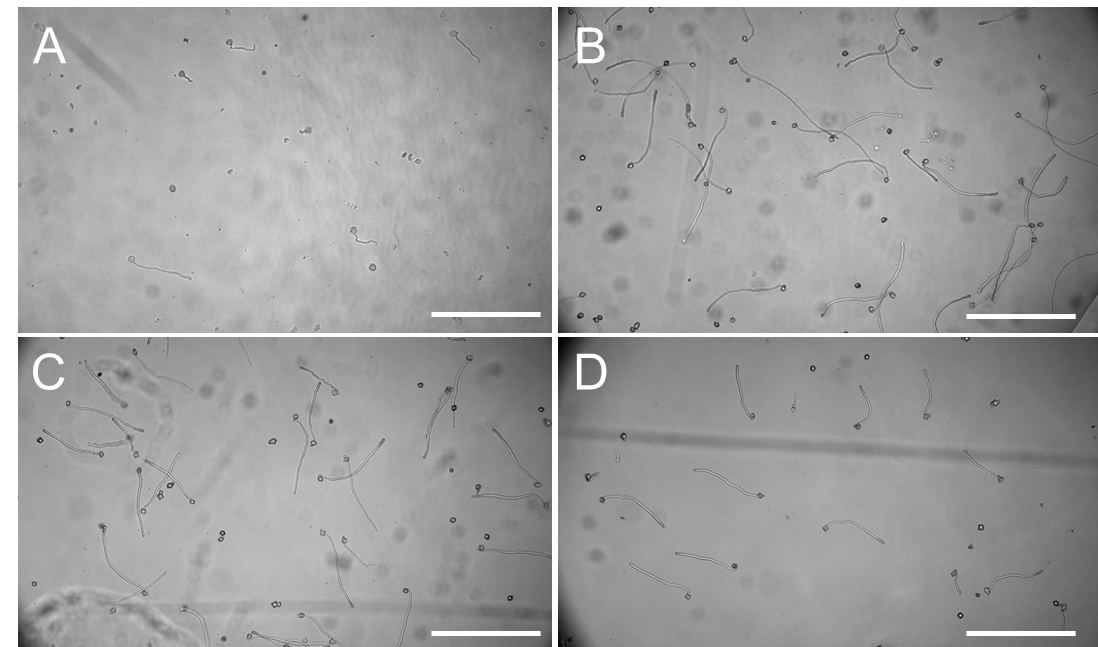


**Figure S1. Impact of different GABA concentration on tobacco pollen tube growth.** Photographs were taken at 6 h cultivation in GM medium. Bars=500 μm.

A.control, without GABA, B. addition with 1.0 mM GABA; B. addition with 10 mM GABA; D. addition with 100 mM GABA.


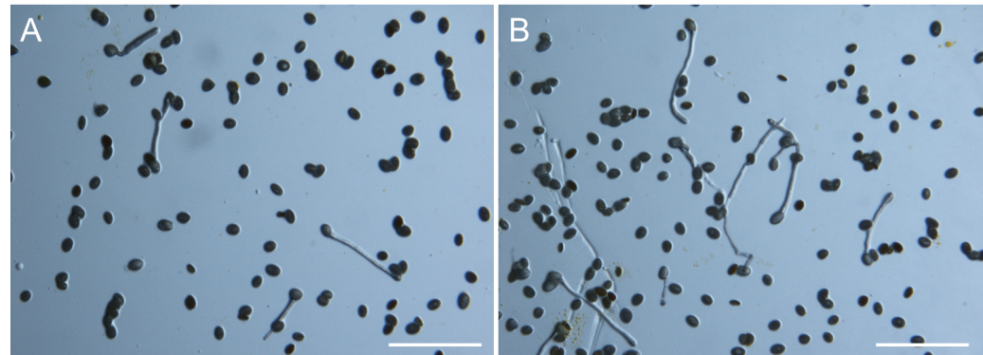


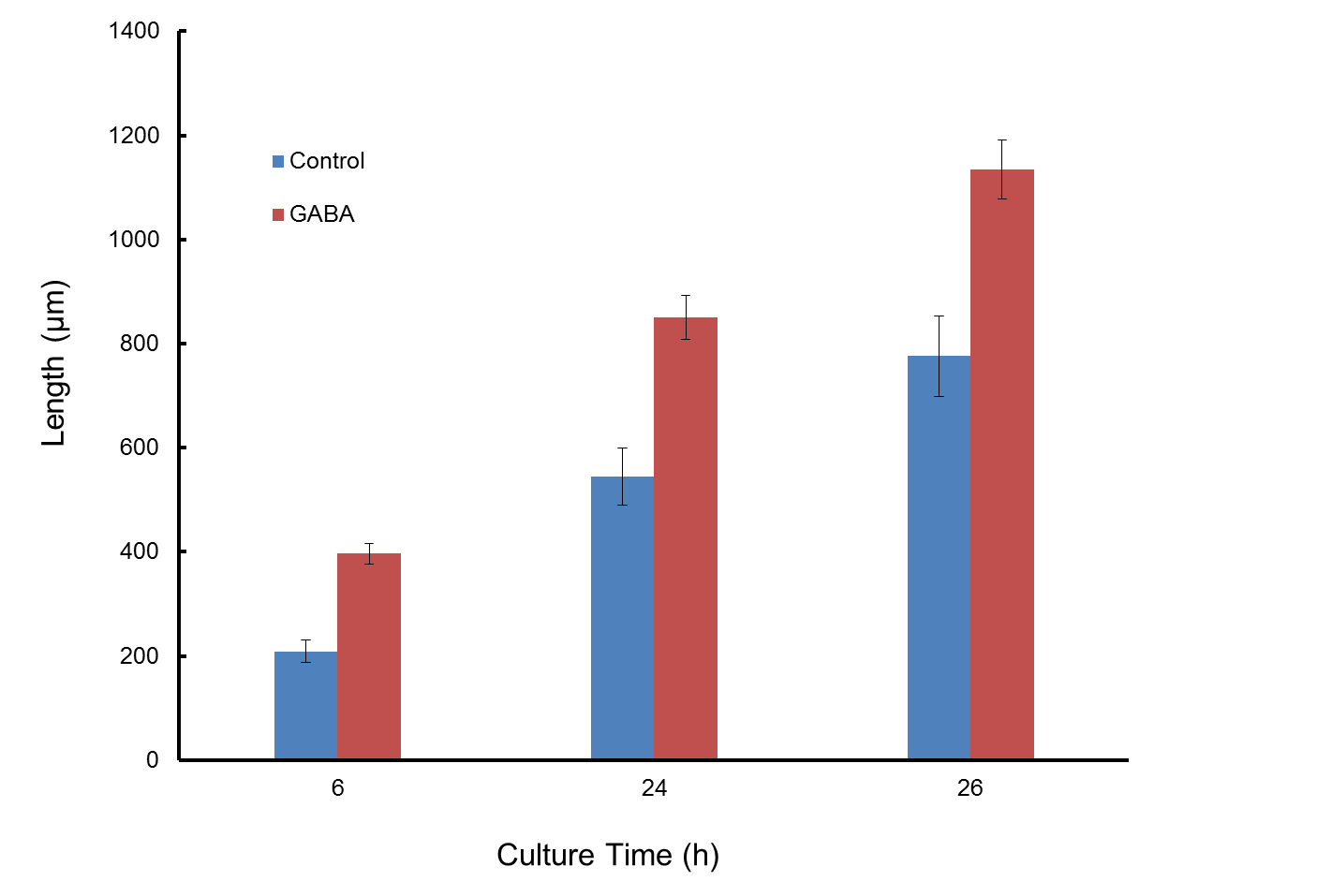


**Figure S2. Effect of GABA on the growth of lily pollen tube growth.**

Pollens of Lily were germinated in medium with and without GABA. A. control, without GABA, B. with GABA of 1 mM GABA. Photographs were taken at 24 h cultivation. Bars=700 μm. Pollen tubes length was measured at different time point of cultivation.

**
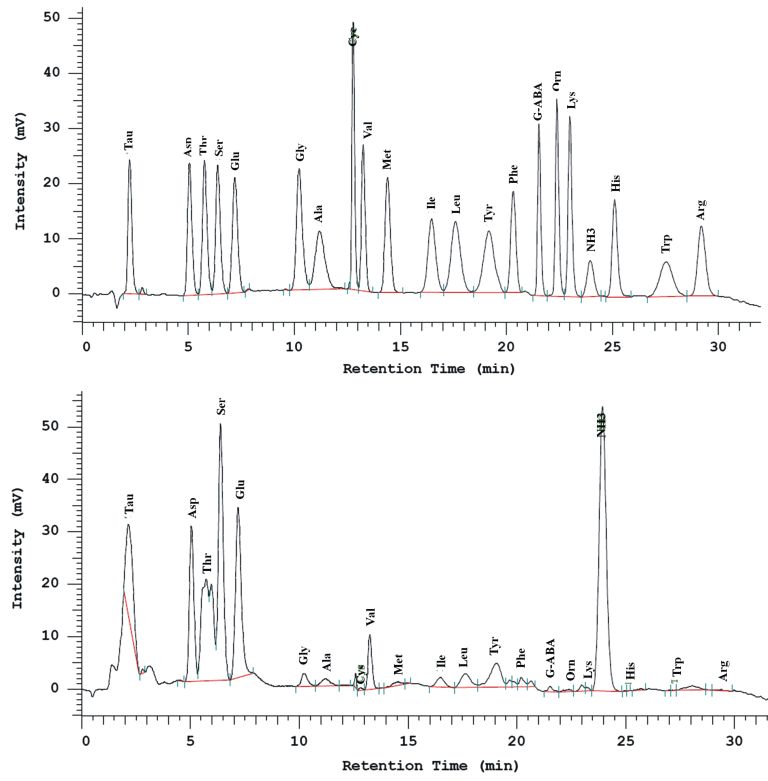
**

**Figure S3.** Content of amino acids was analyzed by L-8800 automatic amino acid analyzer. Data are from 570 nm fluorescent detection channels (proline detected at 440 nm detection is not shown). Upper panel: 20 standard amino acids, Lower panel: free amino acids in typical samples.

**
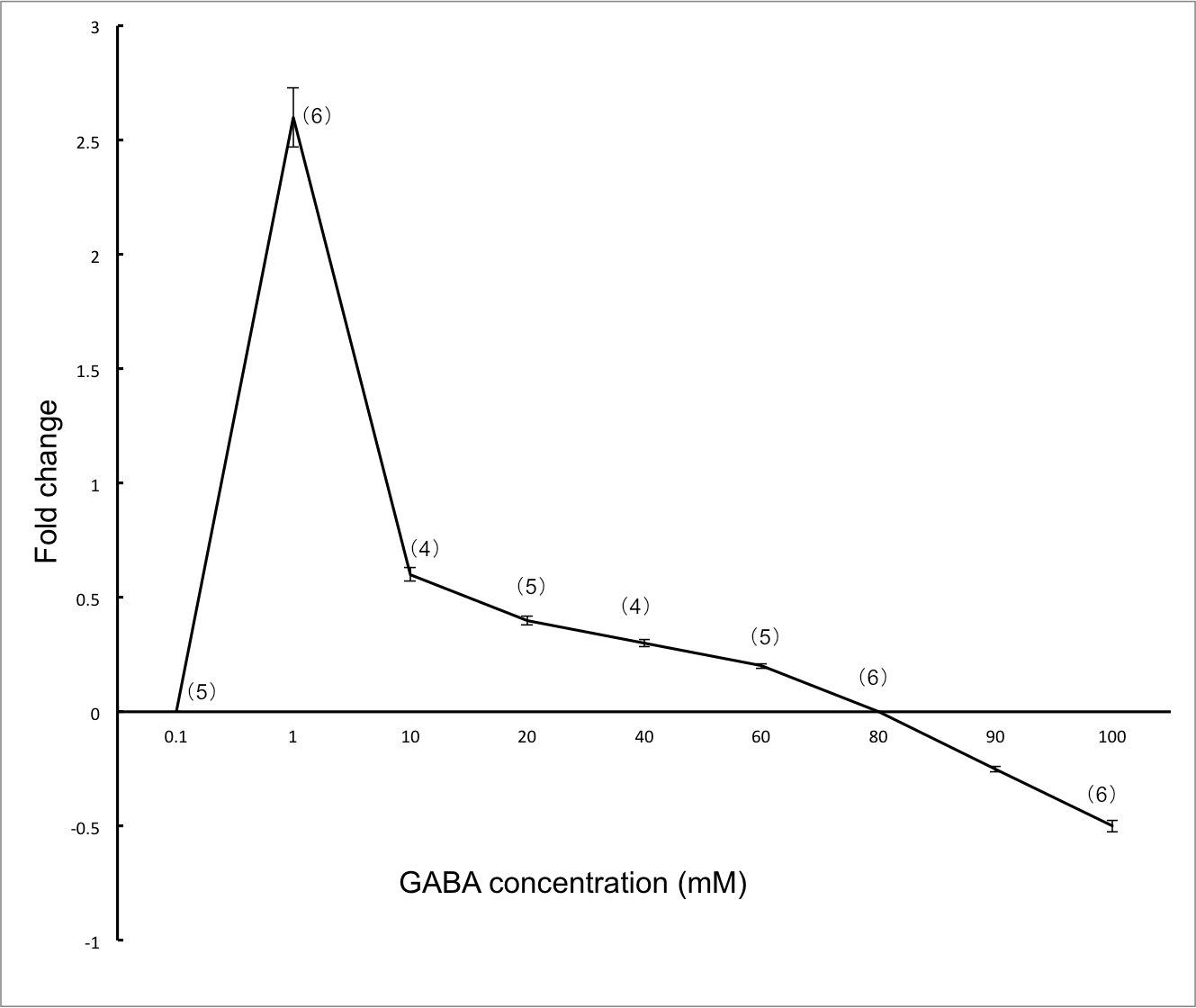
**

**Figure S4. Fold change of whole-cell currents at -200 mV against different GABA concentrations.** N=4-6**.**

**
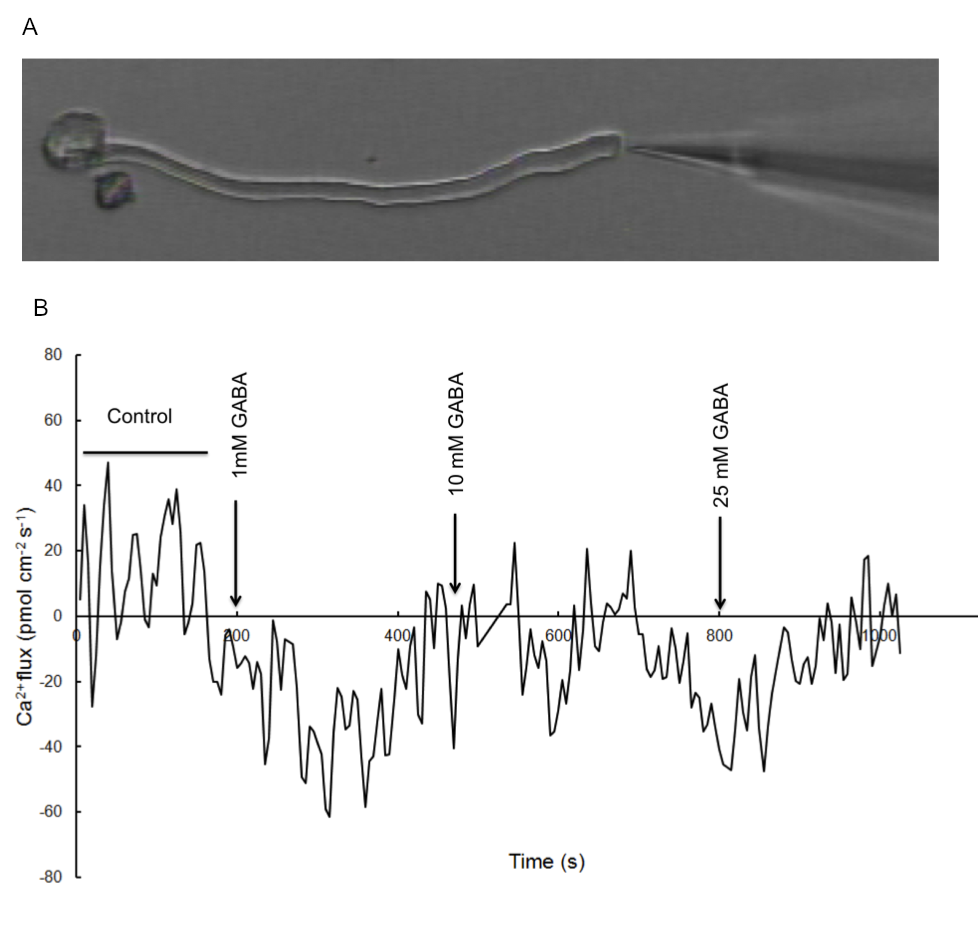
**

**Figure S5. Ca^2+^ oscillations recorded by Non-invasive Micro-test Technology (NMT).** Data from three experimental blocks were best fit using regression of moving average model. **A.** Ca^2+^ specific probe was placed at the tip of tobacco pollen tubes at 10 µm. **B.** Ca^2+^oscillations measurements at different GABA concentration compared to control.

**
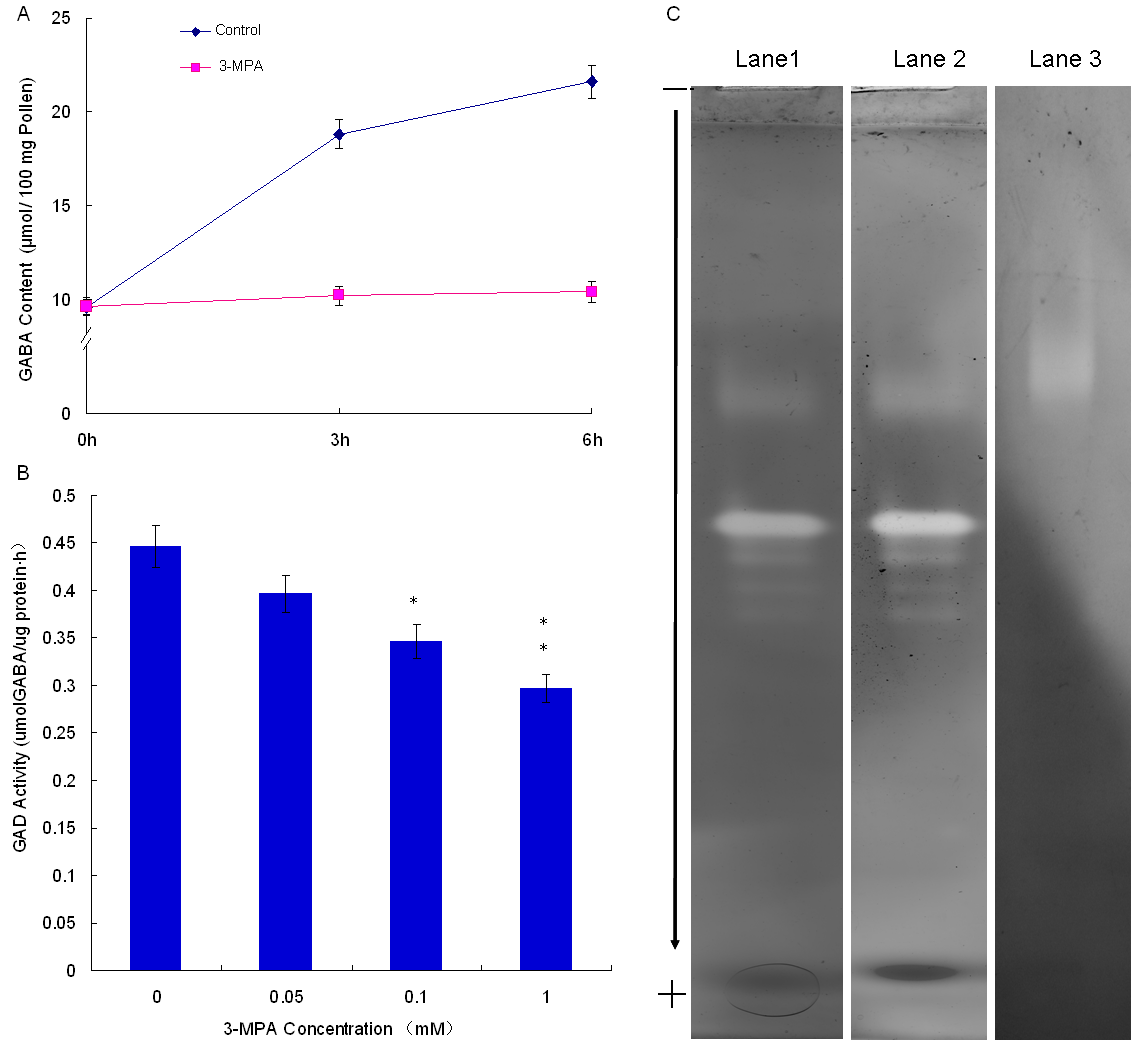
**

**Figure S6. Specific inhibition of 3-MPA on the production of GABA.** A. Effect of the inhibition of 3-MPA on the production of GABA during pollen germination. GABA content was assayed from 100 mg pollen grains, the concentration of 3-MPA was 0.1mM, and error bars represent standard error of means based on three biological replicates. **B.** GAD activity was significantly inhibited by the application of 3-MPA at 0.05, 0.1 and 1.0 mM concentrations. Error bars represent standard error (SE) of means based on three biological replicates. *: *P* <0.05; **: *P* <0.01. **C.** Effect of 3-MPA on the activity of SOD. The concentration of 3-MPA was 0.1mM. Five SOD isoenzymes were displayed on a native activity gel (Lane 1); the same bands were maintained after the treatment with 3-MPA (Lane 2). As a control, only one band (Lane 3) was displayed after the treatment of SOD enzyme inhibitor, H_2_O_2_ (3% concentration).

**
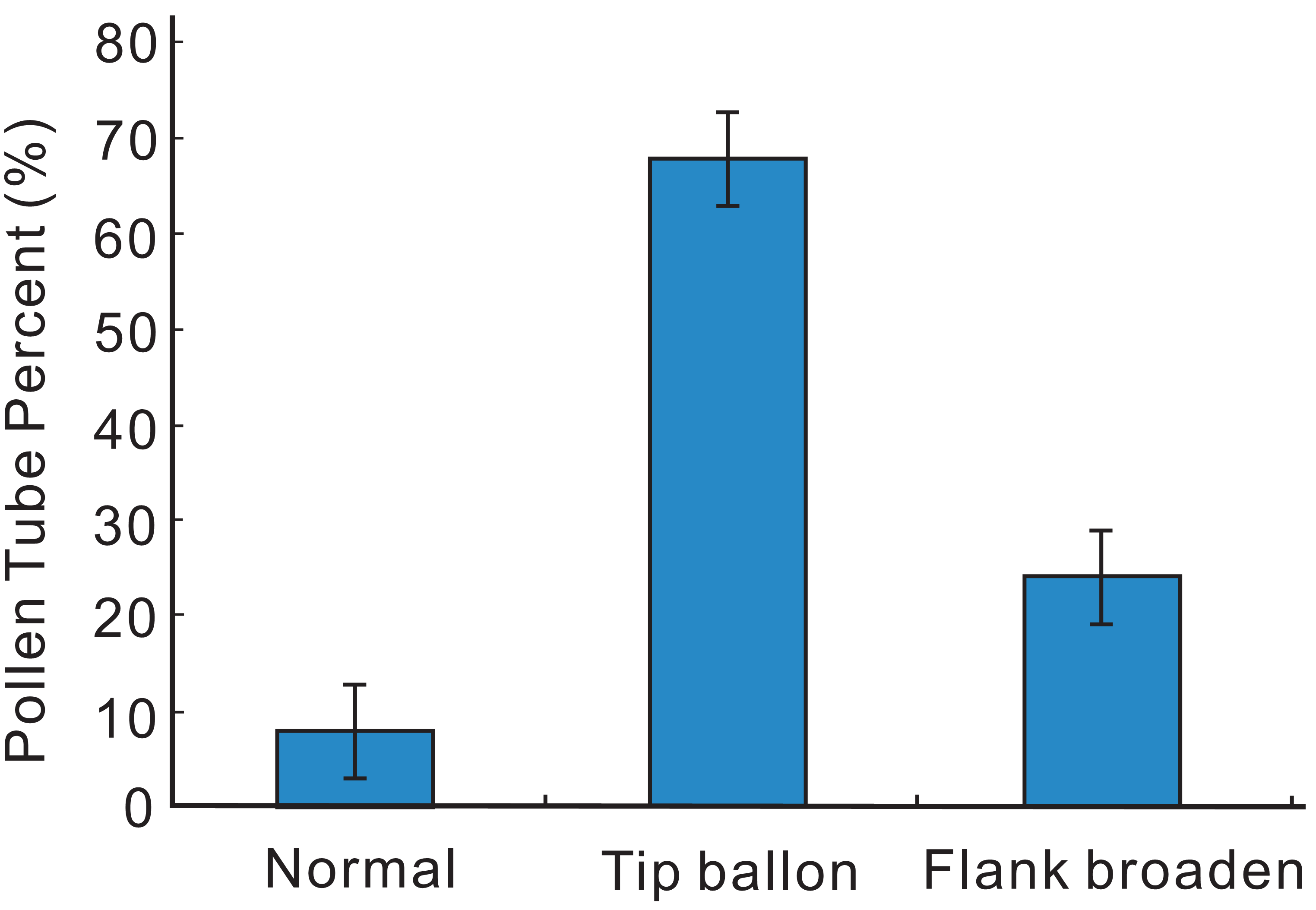
**

**Figure S7.** Percentage of the different abnormal pollen tubes after the application of 1.0 mM 3-MPA application for 3h. Data are means of three biological replicates ±standard error (SE). The abnormal pollen tubes were counted from total 400 pollen tubes. The images were taken by CCD-coupled microscopy.


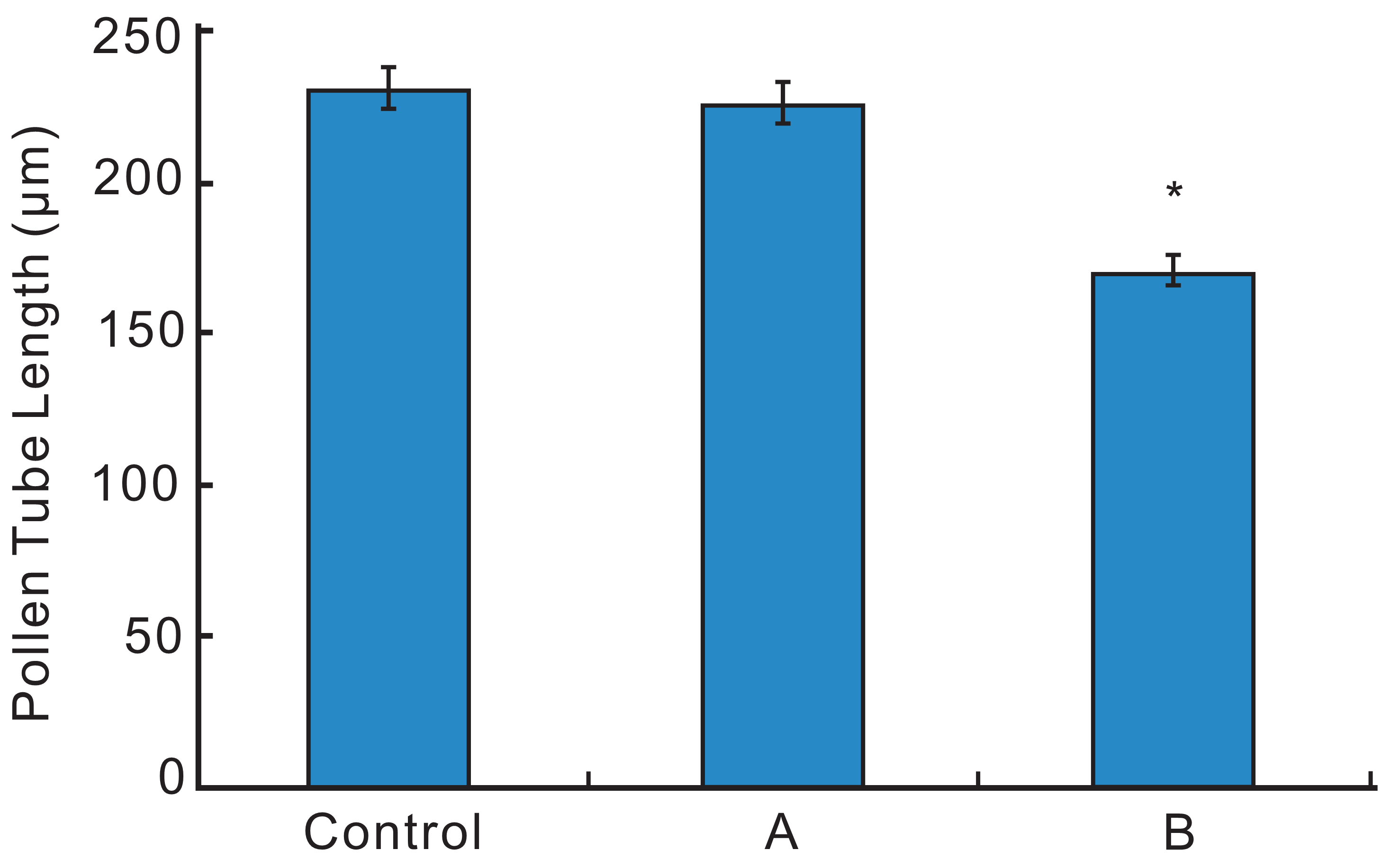


**Figure S8.** The growth inhibition by low concentration of 3-MPA was ameliorated by addition of exogenous GABA. Data are means of three biological replicates ±standard error (SE). Control: germination medium (GM); A: GM + 50 µM 3-MPA + 1.0 mM GABA; B: GM + 50 µM 3-MPA. *: *P* < 0.05. Data were analyzed by one-way ANOVA combined with the post-hoc analysis.

**
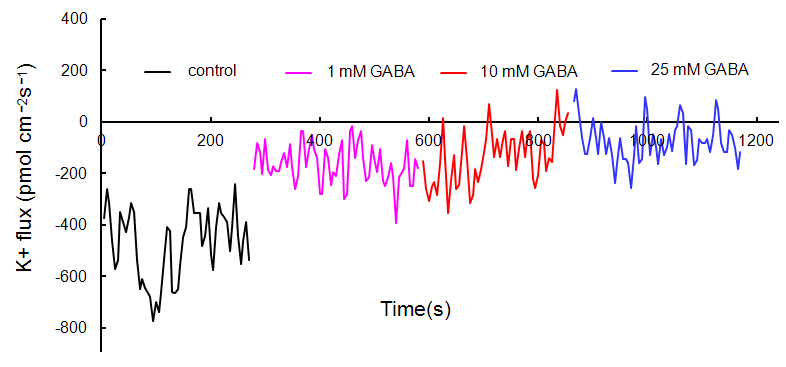
**

**Figure S9. K^+^ oscillation pattern responding to different GABA concentration**. Data from three experimental blocks were best fit using regression of moving average model.

**
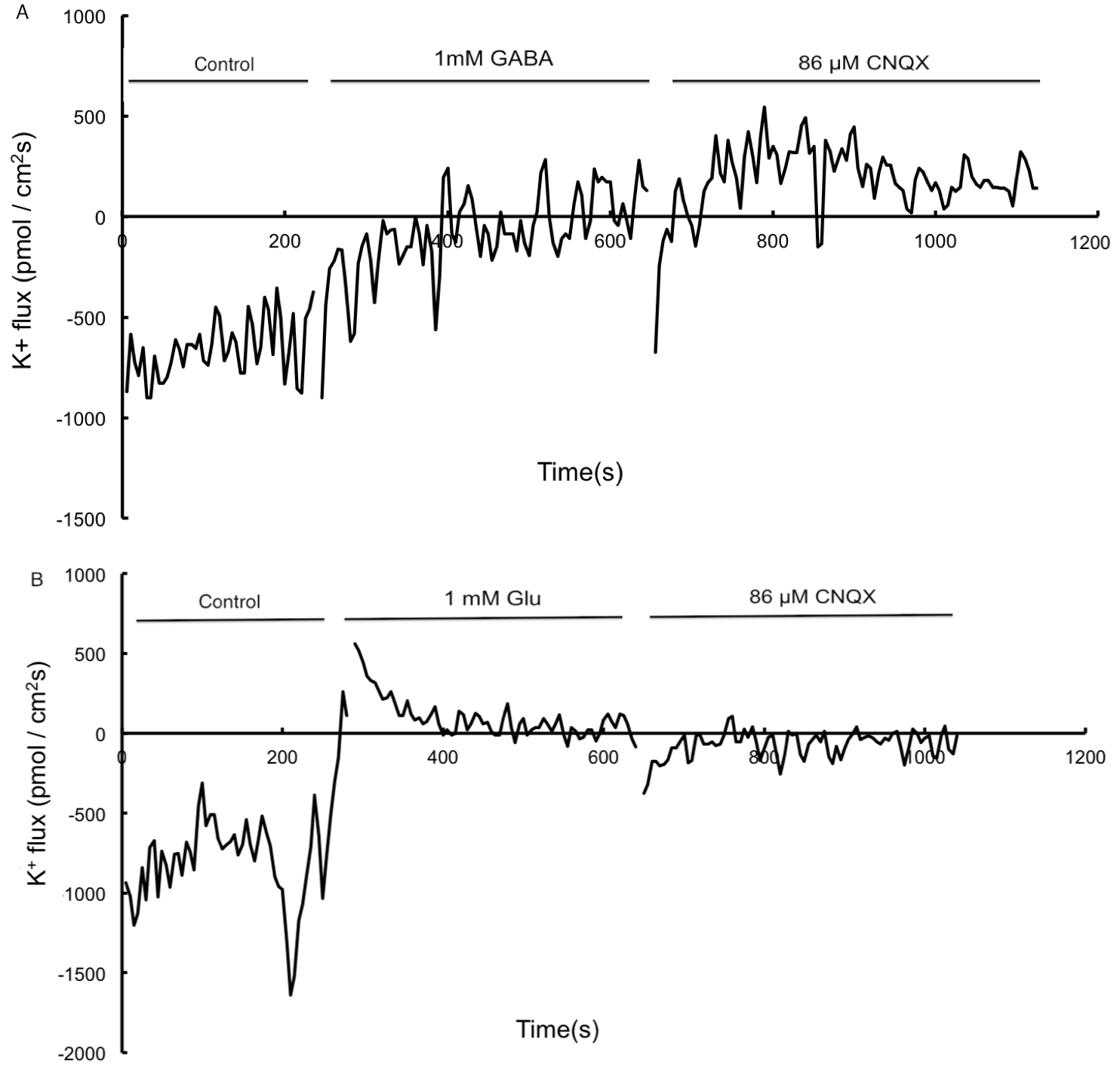
**

**Figure S10. Influence of CNQX after GABA（A）, Glu（B）treatment on K^+^ flux in the tip of tobacco pollen tubes**. Data from three experimental blocks were best fit using regression of moving average model.

**Table S1.**

**Gene-specific primers used in quantitative RT-PCR assay.**

| **Gene**  **Name** | **Access. No**  **In NCBI gene bank** | **Forward Primer** | **Reverse Primer** | **Annealing temperature in PCR** |
| --- | --- | --- | --- | --- |
| *G protein β subunit* | **Z84820** | **AGCTGAAAGAGCGGCATATG** | **CGGAGTTTTACCTTGCGACC** | 53℃ |
| *PLC3* | **EF043044** | **TATCAACTAAGCCACCCAAAGA** | **CTGCTCCGTCATCAGAATCAT** | 53℃ |
| *PLD* | **Z84822** | **GAGCATTTCCCCGACACC** | **CTTCGCATAACAAGGCAACA** | 53℃ |
| *CDPK2* | **AJ344154** | **GGGCTGAAAATGAGCAAGG** | **CCCAACGATGGCACAAGAC** | 55℃ |
| *MAPK* | **X83880** | **TGGAGTTGATTGGCACCC** | **GCGCATCTTCAACGGTAATT** | 52℃ |
| *Rac1* | **AY029330** | **CCCCATACCCATTTCAATCTT** | **CACCATCACCCACCGTCA** | 60℃ |
| *Rab2* | **AF397451** | **AGTTGGCTGGAAGACGCA** | **GAGCAGTTTTGGCAGAGGC** | 55℃ |
| *Rab11b* | **L29269** | **ATGGCAGGTGGGTACAGAGC** | **CGCCGATAGTGGACTTGGA** | 57℃ |
| *RhoGDI2* | **DQ416769** | **GAGAAGGTGGAAGGAGCAGC** | **CTCGGGGATAGGGAGAACA** | 58.5℃ |
| *RhoGAP1* | **DQ813657** | **CCAGGGTGTCTGAATCTAATCG** | **GCCTATCCTTTGAGCGTCC** | 57℃ |
| *GAD1* | **AF352732** | **AGGAAGACTTGCCTGATGAACT** | **CCTCAAAACCCAAGCGAATA** | 53℃ |
| *GAD3* | **AF353615** | **ACAGTTGGTTCATCGGAGGC** | **CCAGTGACAATGTTGGGCTTA** | 55.2℃ |
| ***18s r RNA*** | AJ236016 | AACCATAAACGATGCCGACC | GCCTTGCGACCATACTCCC | 53℃ |

**Video S1** (1.049MB). Time-lapse video showing vesicles trafficking pattern in normal pollen tubes.

**Video S2** (1.370MB). Time-lapse video showing vesicle trafficking pattern in pollen tubes after 1.0 mM 3-MPA treatment.

**Note S1 Calculation of GABA concentration in tobacco style**

To estimate physiological concentrations of GABA in pistil, we transformed the content of GABA in pistil measured by automatic amino acid analyzer into concentration. Since the mass of one gram (g) of tissue is approximately equal to the mass of 1 ml water, we can convert the units of µmol/g into µmol/ mL. With this conversion, the physiological concentration of GABA from stigma to ovary in tobacco pistil ranges from 0.75-4.2 mM.

**Note S2 Specific inhibition of 3-MPA on GAD activity**

We measured the GABA content in pollen tubes as an indicator of GAD inhibition by 3-MPA. During pollen tube growth, the GABA concentration in growing pollen tubes increased from 90 μM to 220 μM after 6 h (Figure. S6A). However, in the presence of 0.1 mM 3-MPA, GABA only increased to ~100 μM after 6 h (Figure. S6A). This result indicates that 3-MPA inhibits the production of GABA during pollen tube growth.

To further test the specificity of 3-MPA inhibition of GAD activity, we employed two methods. First, measurement of *in vitro* GAD activity by the Berthelot color reaction *(6)* showed that steady state levels of GAD activity were high in *in vitro*-grown pollen tubes (Figure. S6B). However, GAD activity was markedly decreased if 3-MPA was included in the reaction mixture and the decrease of GAD activity correlated with the increasing concentrations of 3-MPA (Figure. S6B). This suggests that 3-MPA inhibits the production of GABA by inactivating GAD. In the second method, to test the specificity of 3-MPA’s effect on pollen tube growth, superoxide dismutase (SOD) isoenzyme activity, present at high levels in tobacco pollen (Wang et al., 2009), was assessed as a negative control by native polyacrylamide gel electrophoresis. In this assay, Nitroblue Tetrazolium (NBT) staining detected five SOD isoenzymes (Figure. S6C). After 3-MPA treatments, all five SOD isoenzymes could still be detected (Figure. S6C). However, they were absent in pollen tubes treated with H_2_O_2_, an inhibitor of SOD isoenzymes. These results indicated that 3-MPA specifically inhibited the activity of GAD.

**Note S3 K^+^ flux responds to GABA**

Known from research in animal cells, activation of Ca^2+^ channel coupled with K^+^ channels in responding to GABA signal. To detect this possibility, we further monitor K^+^ flux using K^+^-selective vibrating probe approach. K^+^ demonstrated influx oscillation pattern in the pollen tubes grew *in vitro*, however, addition of GABA decreased K^+^ influx and has the trend to reverse the K^+^ influx to outward flux with GABA concentration increasing (Figure. S9). GABA treatment caused the decrease of K^+^ influx, indicating that K^+^ channel is reversely controlled by GABA signals. Subsequently, the addition of CNQX caused further K^+^ outflux (Figure. S10A). This pattern is different from that caused by glutamate, which was inhibited by the addition of CNQX, a putative inotropic glutamate-receptor-like inhibitor (Michard et al., 2011) (Figure. S10B). This indicates the characteristics of Ca^2+^ influx coupled with K^+^ outward flux is different from that of glutamate, and putative metabolic GABA_B_ receptors may exit and involve in response to GABA signal.

**Supplementary References**

Baum, G., Lev-Yadun, S., Fridmann, Y., Arazi, T., Katsnelson, H., Fromm, H. (1996). Calmodulin binding to glutamate decarboxylase is required for regulation of glutamate and GABA metabolism and normal development in plants. *EMBO J*. **15**, 2988-2996.

Chen, Y., Baum, G., Fromm, H. (1994). The 58-kilodalton calmodulin-binding glutamate decarboxylase is a ubiquitous protein in *Petunia* organs and its expression is developmentally regulated. *Plant Physiol*. **106**, 1381-1387.

Johnson, B.S., Singh, N.K., Cherry, J.H., Locy, R.D. (1997).Purification and characterization of glutamate decarboxylase from cowpea. *Phytochem*. **46,** 39-44

Liu, C.L., Zhao, L., Yu, G.H. (2011). The dominant glutamic acid metabolic flux to produce γ-amino butyric acid over proline in *Nicotiana tabacum* leaves under water stress relates to its significant role of antioxidant activity. *J. Integr. Plant Biol.* **53** (8): 608–618.

Martinez-Garcia, J.F., Monte, E., and Quail, P.H. (1999). A simple, rapid and quantitative method for preparing *Arabidopsis* protein extracts for immunoblot analysis. *Plant J*. **20**, 251-257.

Michard, E., Lima, P.T., Borges, F., Silva, A.C., Portes, M.T., Carvalho, J.E., Gilliham, M., Liu, L.H., Obermeyer, G., Feijó, J.A.(2011).Glutamate receptor-like genes form Ca^2+^ channels in pollen tubes and are regulated by pistil D-serine. *Science* **332**, 434-437.

Snedden, W.A., Koutsia, N., Baum, G., Fromm, H. (1996). Activation of a recombinant petunia glutamate decarboxylase by calcium/calmodulin or by a monoclonal antibody which recognizes the calmodulin-binding domain. *J. Biol. Chem.* **271**, 4148-4153.

Wang, J., Liu, X. Q., Yu, G. H. (2009). Identification of superoxide dismutase isoenzymes in tobacco pollen. *Frontier Biol China*.**2**, 425-442.
